# Estimation of chemical and physical effects of cavitation by analysis of cavitating single bubble dynamics

**DOI:** 10.1016/j.ultsonch.2021.105677

**Published:** 2021-07-23

**Authors:** Ajinkya V. Pandit, Varaha P. Sarvothaman, Vivek V. Ranade

**Affiliations:** a School of Chemistry and Chemical Engineering, Queen’s University, Belfast, UK; b Bernal Institute, University of Limerick, Limerick, Ireland

**Keywords:** Hydrodynamic - acoustic cavitation, Hydroxyl radicals, Cavitation jets, Localised energy dissipation

## Abstract

Cavitation events create extreme conditions in a localized ‘bubble collapse’ region, leading to the formation of hydroxyl radicals, shockwaves and microscopic high-speed jets, which are useful for many chemical and physical transformation processes. Single bubble dynamics equations have been used previously to investigate the chemical and physical effects of cavitation. In the present study, the state of the art of the single bubble dynamics equations was reviewed and certain noteworthy modifications were implemented. Simulations reaffirmed previously reported collapse temperatures of the order ~5,000 K and collapse pressures well over ~1,000 bar under varying operating conditions. The chemical effects were assessed in terms of the hydroxyl radical generation rate (OHG), calculated by applying the minimization of the Gibb’s Free Energy method using simulated collapse conditions. OHG values as high as 1x10^12^•OH molecules per collapse event were found under certain operating conditions. A new equation was proposed to assess the physical effects, in terms of the impact pressure of the water jet - termed as the jet hammer pressure (JHP), formed due to the asymmetrical collapse of a bubble near a wall. The predicted JHP were found to be within a range of ~100 to 1000 bar under varying operating conditions. Important issues such as the onset of cavitation and chaotic solutions, for a cavitating single bubble dynamics were discussed. The Blake threshold pressure was found to be a sufficient criterion to capture the onset of cavitation. The impact of key operating parameters on the chemical and physical effects of cavitation were investigated exhaustively through simulations, over the parameter ranges relevant to acoustic and hydrodynamic cavitation processes. Presented methodology and results will be useful for optimisation and further investigations of a broad range of acoustic and hydrodynamic cavitation-based applications.

## Highlights

• Comprehensive model for cavitating single bubble dynamics (code on GitHub).
• Exhaustive simulations spanning an operating parameter space of practical interest.
• Quantitative information on physical and chemical effects of cavitation.
• Results will be useful for a broad range of cavitation applications.

## Graphical abstract

**Figure:**
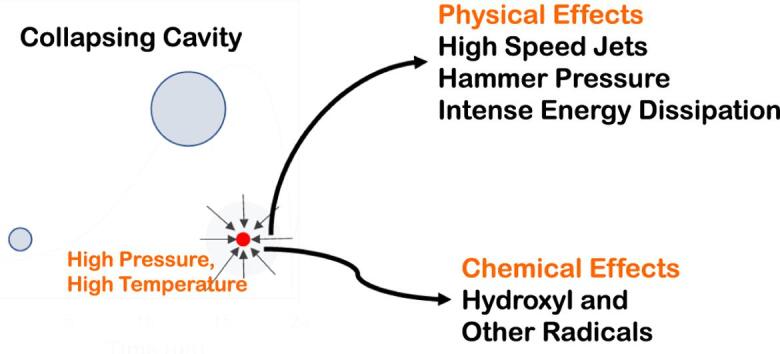

## Nomenclature

a: Pressure correction factor for Van der Waal’s equation, $\frac{{Pa-m}^{6}}{{\mathrm{kmol}}^{2}}$
b: Volume correction factor for Van der Waal’s equation, $\frac{m^{3}}{\mathrm{kmol}}$
c: Velocity of sound, m/s
C: Concentration, $\frac{No.ofmolecules}{m^{3}}$
$C_{v}$: Heat capacity at constant volume, $\frac{J}{K*No.ofmolecules}$
$C_{p}$: Heat capacity at constant pressure, $\frac{J}{K*No.ofmolecules}$
D: Diffusion coefficient,$m^{2}/s$
f: Degrees of freedom, -
F: Driving Frequency, Hz
h: Molecular enthalpy of i^th^ species at the interface, $\frac{J}{\mathrm{No}.ofmolecules}$
k: Boltzmann’s constant, $\frac{J}{K*No.ofmolecules}$
$l_{\mathrm{diff}}$: Diffusion length for mass transfer, m
$l_{\mathrm{th}}$: Diffusion length for heat transfer, m
m: Mass of a molecule, $\frac{\mathrm{kg}}{\mathrm{No}.ofmolecules}$
MW: Molecular weight, kg/kmol
$N_{A}$: Avagadro’s constant (6.023x10^26^),$\frac{\mathrm{No}.ofmolecules}{\mathrm{kmol}}$
N: Number of molecules, No.ofmolecules
$N_{C}$: Number of components in the bubble, -
$N_{\theta }$: Number of components of characteristic vibrational temperature, -
P: Pressure, Pa
r: Radial co-ordinate, -
R: Radius of the bubble,m
s: Velocity of the bubble wall, m/s
t: Time, s
T: Temperature, K
V: Volume, $m^{3}$
x: Mole fraction, -
v: Velocity, m/s
K: Kinetic energy per unit mass,$m^{2}/s^{2}$
$l_{\mathrm{eddy}}$: Average length of eddy, m
${\Delta P}_{\mathrm{cavity}}$: Average pressure due to cavity expansion and contraction, Pa
E: Energy, J
z: Constant for jet velocity calculations, -

**Greek Symbols**
α: Molecular diameter for species i, m
β: Correction factor in the calculation for thermal conductivity, -
δ: Variable for the calculation of heat capacity, -
∊: Minimum of the pair potential energy, J
η: Viscosity,Pas
κ: Thermal diffusivity, $m^{2}/s$
λ: Thermal conductivity, $W/\left(mK\right)$
$\emptyset_{1}$: Keller Miksis Correction Factor 1, -
$\emptyset_{2}$: Keller Miksis Correction Factor 2, -
$\emptyset_{3}$: Keller Miksis Correction Factor 3, -
ν: Kinematic viscosity of liquid medium, $m^{2}/s$
ω: Angular frequency – acoustic cavitation, rad/s
$\Omega_{i,j}^{\left(1,1\right)}$: Collision integral for binary diffusion coefficient calculations, -
$\Omega_{i,i}^{\left(2,2\right)}$: Collision integral for viscosity calculations for pure substance, -
ρ: Density, $\mathrm{kg}/m^{3}$
$\rho_{\mathrm{mix}}$: Density of number of molecules, $\frac{No.ofmolecules}{\mathrm{gmol}}$
σ: Surface tension, N/*m*
Γ: Reduced molecular mass, kg/kmol
ψ: Lennard-Jones force constant, K
Ψ: Correction term contribution for $C_{v,mix}^{*},J/K$
θ: Characteristic vibrational temperature, K
ε: Instantaneous turbulent energy dissipation rate per unit mass, $\frac{J}{kg-s}$
μ: Dynamic viscosity of liquid medium, Pa-s
γ: Normalized stand-off parameter, -

**Subscripts**
0: Initial Condition
L: Pertaining to the bulk medium - liquid
i,j: Indices to represent species
w: Water
i≠w: All species except water
t: Total number
v: Pertaining to vapour pressure
mix: Pertaining to a mixture
v: Pertaining to vapour pressure
volatile: Volatile component
max: Maximum
C: Pertaining to cavity collapse conditions
∞: Unbounded liquid medium
J: Cavitation Jet
S: Shockwave
LC: Laser Induced Cavitation
H: Water Hammer
A: Amplitude of oscillations

**Superscripts**
B: Pertaining to bubble internal contents
∞: Pertaining to far field bulk medium conditions - liquid
i: Pertaining to the liquid-bubble interface
∗: Corrected term
': Reduced/Normalized Term

**Abbreviations**
AC: Acoustic cavitation
HC: Hydrodynamic cavitation
RP: Rayleigh-Plesset
KM: Keller-Miksis
EoS: Equation of State
VdW: Van der Waal’s
IG: Ideal Gas
MGFE: Minimization of Gibbs free energy
LC: Laser-induced Cavitation
OHG: •OH Generation
JHP: Jet Hammer Pressure
JV: Jet Velocity
TEDR: Turbulent Energy Dissipation Rates

## 1 Introduction

Cavitation first studied by Lord Rayleigh was identified as the cause for the damage to ship propellers rotating at very high speeds and thus posed a limitation for further technological improvements [1], [2]. Although cavitation is still a cause for concern for high-speed pumps [3], propellers [1], [2] and hydraulic machinery [4], numerous beneficial applications of cavitation have also been discovered, which harness the chemical and/or the physical effects of cavitation [5]. Cavitating bubbles have been reported to produce extremely high temperatures (of the order ~ 5,000 K) and pressures (well over 1,000 bar) in the interior of the bubble [6], which under certain conditions may even lead to the emission of light (sonoluminescence) [7], [8]. Under such extreme conditions, cavitation in water produces hydroxyl radicals (•OH), which have very high reaction rates, making them very potent oxidising agents [9]. This chemical effect of cavitation has been reported to be highly effective as an advanced oxidation process for the potentially chemical free removal of several recalcitrant chemicals [4], [10], [11]. Cavitation events occurring closer to a wall, have been reported to lead to the formation of shockwaves and high-speed water jets (piercing through the bubbles) which damage the wall material [12]. Impulses with extremely high pressure are continually generated during cavitation which cause fatigues stresses and eventually fragmentation [13]. This physical effect of cavitation has been shown to be beneficial for various chemical free pre-treatments like for biomass valorisation [14], [15], crystallisation [16] or emulsification [17].

Single bubble dynamics equations are widely used to investigate cavitation processes. The single bubble dynamics equation first proposed by Lord Rayleigh [1], [2], known popularly as the Rayleigh-Plesset (RP) equation, was a liquid side momentum balance allowing for a moving boundary at the bubble interface and enabled the tracking of the bubble radius response to an oscillating pressure field. This equation was subsequently modified to include the complete effects of surface tension and the principal effects of viscosity to give the Gilmore’s equation [18] and later to accurately characterise the bubble response to large pressure oscillations as given by the Keller-Miksis (KM) equation [19]. The latter equation is usually the equation of choice for the solution of the single bubble dynamics problems in recent studies [20], [21], [22], [23]. It is worthwhile to note that all these equations deal with a single spherically symmetric collapsing bubble. It has been reported that cavitation closer to a wall leads to more complex dynamics of asymmetric collapse [24], leading to jet formation [12] and decreasing the overall chemical effect of cavitation due to milder collapse conditions. More sophisticated interface tracking models using finite volume and volume of fluid methods, accounting the nonlinear compressibility effects for an asymmetrically collapsing bubble, have also been proposed recently [25]. However, these models do not account for the bubble internal dynamics which govern the chemical effects of cavitation.

Model developments coupling the single bubble dynamic equations to important process physics such as the diffusion of water molecules, heat conduction and equation of state to track bubble internal conditions were driven largely by investigations into sonoluminescence. The single bubble dynamics models coupled with a detailed chemical kinetics model, enabled the first predictions of the hydroxyl radical generation rate due to cavitation events [7], [20] and have also been used recently to provide further insights into the chemical effects of cavitation [26], [27]. Alternatively, such predictions could also be made by using the Gibbs free energy minimization method [28], [29], [30]. Due to the extremely short lifespan of the hydroxyl radicals [9] and several uncertainties surrounding cavitation, model predictions could only be indirectly validated by inferring the associated decrease in the concentration of target chemicals. Often the non-selective nature of hydroxyl radicals led to the formation of a wide array of oxidation products especially for complex chemical species, further complicating the accurate characterisation of hydroxyl radicals [31]. Chemical reaction engineering incorporating the results from single bubble dynamics model have since been proposed to model the chemical effects of cavitation [28], [29], [30].

In applications benefitting from the physical effects of cavitation, it is important to estimate a ‘cavitation potential’ for quantifying process performance. However, there is no consensus with regards to quantitatively predicting the physical effects of cavitation [4]. The physical effects of cavitation have been attributed to shockwave formation due to bubble collapse [12], [32], viscous dissipation due to an oscillating bubble (intense shear generated due to the sudden bubble expansion–contraction) [33] and the jet formation due to asymmetric collapse [12], [34]. Philipp & Lauterborn (1998) [12] reported through high-speed visualisations and analysis of damage patterns on aluminium plates that both shockwave and jet formation contributed to cavitation damage/ physical effects and that cavitation nearer to a wall led to an asymmetric collapse and the associated jet formation. This finding was also supported by Supponen et. al. (2017) [32] who further reported a decreasing intensity in the measured shockwave energy for cavitation events closer to a wall through hydrophone measurements.

Modelling approaches to predict the damage due to cavitation have been scarce. Supponen et. al. (2017) [32] provided semi-empirical relationships to correlate the initial bubble energy to the energy dissipated as shockwaves and the distance from the wall. Versluis et. al. (2000) [35] coupled the single bubble dynamics model with the equation for sound propagation in a liquid to describe the bubble collapse effect in terms of the pressure at a certain distance away from the cavitation event. Pecha et. al. (2000) [36] successfully predicted the shockwave pressure (obtained by analysing the shockwave velocity recorded using a streak camera) at the cavitation source by using a single bubble dynamics model. The experimental results indicated a rapid decrease in the shockwave pressure as a function of the distance from the cavitation event, however, these were not modelled. Peng et. al. (2018) [34] used the single bubble dynamics model like the approach of Versluis et. al. (2000) [35] to investigate the potential of self-resonating cavitating jets in breakage of rocks. However, all these approaches using the single bubble model did not account for the complex physics of asymmetric collapse [24].

In the present study, first various key aspects (model formulation, model verification, onset of cavitation, chaotic behaviour) of a cavitating single bubble dynamics were investigated using a single bubble model. The model equations describing single bubble dynamics were modified by relaxing certain assumptions and several nuances surrounding the topic were addressed. The method for estimating the hydroxyl radical generation rate in general followed previous approaches – using the minimization of Gibbs free energy method, albeit using the modified model equations proposed in the present study. A new equation was derived to estimate the physical effects of cavitation arising due to the jet formation caused by asymmetric bubble collapse, in terms of the parameter - jet hammer pressure, which was argued to be useful from a practical standpoint. The operating parameter ranges for typical applications of both acoustic cavitation (AC) and hydrodynamic cavitation (HC), were clearly identified and estimates for the hydroxyl radical generation rate and the jet hammer pressure were obtained covering the respective operating parameter space. Based on simulation results, key trends in terms of the chemical and physical effects of cavitation were concisely summarized which will be useful to improve the performance of cavitation processes. The presented results and comprehensive information included in the Supporting Information will enable use of the microscale single bubble dynamics model with other macro-scale reaction engineering models.

## 2 Mathematical model

### 2.1 Equations governing single bubble dynamics

The Keller-Miksis (KM) equation includes the effects of various non-idealities [19] whilst retaining a relative simplicity and has been a popular choice to model single bubble dynamics [20], [21], [22], [23]. In the present study the KM equation given by Equation (1), was used to resolve the bubble dynamics. The KM equation is a second order differential equation and may be reduced to two first order simultaneous ordinary differential equations as given by Equations. (2), (3).

(1) $$\emptyset_{1}R\frac{d^{2}R}{dt^{2}}+\frac{3}{2}\emptyset_{2}{\left(\frac{\mathrm{dR}}{\mathrm{dt}}\right)}^{2}=\frac{\emptyset_{3}}{\rho_{L}}\left(P^{B}-P_{L}^{\infty }\left(t\right)\right)+\left(\frac{R}{\rho_{L}c_{L}}\right)\frac{{\mathrm{dP}}^{B}}{\mathrm{dt}}-\left(\frac{4\nu_{L}}{R}\right)\frac{\mathrm{dR}}{\mathrm{dt}}-\frac{2\sigma_{L}}{\rho_{L}R}$$

(2) $$\frac{\mathrm{dR}}{\mathrm{dt}}=s$$

(3) $$\frac{\mathrm{ds}}{\mathrm{dt}}=\left(\frac{\emptyset_{3}\left(P^{B}-P_{L}^{\infty }\left(t\right)\right)}{\emptyset_{1}\rho_{L}R}\right)+\left(\frac{1}{\emptyset_{1}\rho_{L}c_{L}}\right)\frac{dP^{B}}{\mathrm{dt}}-\left(\frac{4\nu_{L}s}{\emptyset_{1}R^{2}}\right)-\left(\frac{2\sigma_{L}}{\emptyset_{1}\rho_{L}R^{2}}\right)-\left(\frac{3\emptyset_{2}s^{2}}{2\emptyset_{1}R}\right)$$

(4) $$\emptyset_{1}=1-\frac{s}{c_{L}}\emptyset_{2}=1-\frac{s}{3c_{L}}\emptyset_{3}=1+\frac{s}{c_{L}}$$

### 2.2 Equation of state

To model the gas phase dynamics inside the bubble as a response to the changing bubble radius, a gas phase equation of state (EoS) needs to be solved simultaneously with the Equations (1), (2), (3), (4). The bubble interior temperature and pressure conditions at collapse, given by a suitable EoS, are required to estimate key performance parameters such as the hydroxyl radical generation rate and the shockwave pressure. Previous studies have considered the Sauve-Redlich-Kwong EoS [7], the Van der Waal’s (VdW) EoS [20], [21] or some variations thereof [22], [23], and the Ideal Gas (IG) EoS [21]. It was previously reported that the choice of the EoS did not affect the basic physics of cavitation but only affected the final quantitative values [7], [21]. At the point of bubble collapse, internal temperatures and pressures of the order of ~ 10,000 K and ~ 1,000 bar have been reported [6] and hence, under such extreme conditions, the validity of any EoS is not beyond reproach. If studies dealing with supercritical phases are any indication, predictions using the various considered EoSs were seen to converge [37] at the high pressure and temperature conditions (~1,000 K and ~ 100 bar). However, preliminary simulations revealed notable deviations in collapse conditions only for very high-pressure amplitudes. Hence, in the present study, the VdW EoS as given by Equation (5) was used. The equation for the rate of change of bubble interior pressure required for the closure of Equation (3) is given in Section A1 of the Supporting Information along with the derivation.

(5) $$P^{B}=\frac{N_{t}^{B}kT^{B}}{\frac{4\pi }{3}R^{3}-\frac{N_{t}^{B}}{N_{A}}b}-a\frac{{\left(\frac{N_{t}^{B}}{N_{A}}\right)}^{2}}{{\left(\frac{4\pi }{3}R^{3}\right)}^{2}}$$

(6) $$a=\sum_{i}a_{i}{x_{i}^{B}}^{2};b=\sum_{i}x_{i}^{B}b_{i}$$

### 2.3 Inclusion of water vapour

The mass transfer of water molecules from the surrounding liquid into the cavitation bubble was neglected [20] until Moss et. al. (1999) [38] experimentally demonstrated that water vapour played a significant role in determining cavity collapse conditions [7], [20]. Thereafter the seminal papers by Storey et. al. (2000) [7] and Toegel et. al. (2000) [20] supported the conclusions of Moss et. al. (1999) [38] regarding the inclusion of water vapour through simulations and by considering a diffusion limited mass transfer model. Following these publications, subsequent studies dealing with the single bubble cavitation models [22], [23], [29] usually include the diffusion limited mass transfer of water, largely following the treatment by Toegel et. al. (2000) [20]. In the model reported by Storey et. al. (2000) [7] the radial variation in the concentration (change in concentration inside the bubble along the radius assuming radial symmetry) of chemical species was considered along with the diffusion of water vapour from the bubble surface. Thereafter, Toegel et. al. (2000) [20] demonstrated that similar predictions as Storey et. al. (2000) [7], in terms of the water content and the bubble radial profile, may be obtained by assuming the bubble internal contents to be perfectly mixed (homogeneous).

Following upon this thread, in the present study, a diffusion limited mass transfer model for water was considered and it was further assumed that the bubble internal contents were perfectly mixed (homogeneous). To calculate the diffusive flux at the surface, the interfacial gas film was assumed to be in equilibrium with the liquid and the flux was calculated using the gradient of concentration as given by Equation (7). The thickness of the diffusion layer was calculated as per the model proposed by Toegel et. al. (2000) [20], given by Equation (8). The rest of the parameters required for the closure of the mass transfer model were calculated as per the molecular theory of thermodynamics [39], [40] and are shown in Section A2 of the Supporting Information.

(7) $$\frac{dN_{i}^{B}}{\mathrm{dt}}=-4\pi R^{2}D_{i}{\left(\frac{\partial C_{i}}{\partial r}\right)}_{r=R}=4\pi R^{2}D_{i}\left(\frac{C_{i}^{i}-C_{i}^{B}}{l_{\mathrm{diff}}}\right)$$

(8) $$l_{\mathrm{diff}}=\min\left(\sqrt{\frac{RD_{i}}{\left|s\right|}},\frac{R}{\pi }\right)$$

### 2.4 Heat transfer

The present study follows the heat transfer model reported by Toegel et. al. (2000) [20] with a noteworthy exception described later. The heat balance equation as given by Equation (9), is the balance for the internal energy of bubble contents and includes the terms for the work transferred between the liquid and bubble during expansion–contraction, the heat conduction between the bubble and liquid at the interface and the enthalpy of the species diffusing in/out of the bubble. The equation for the internal energy with certain mathematical manipulations may be changed to obtain the equation for temperature as given by Equation (10).

(9) $$\frac{d}{\mathrm{dt}}\left(\sum_{i=1}^{N_{c}}C_{v,i}N_{i}^{B}T^{B}\right)=\left[\left(-4\pi R^{2}\lambda {\left(\frac{\partial T}{\partial r}\right)}_{r=R}\right)-\left(4\pi R^{2}P^{B}\frac{\mathrm{dR}}{\mathrm{dt}}\right)+\left(\sum_{i=1}^{N_{c}}h_{i}\frac{dN_{i}^{B}}{\mathrm{dt}}\right)\right]$$

(10) $$\frac{dT^{B}}{\mathrm{dt}}=\frac{1}{C_{v,mix}^{*}}\left[\left(-4\pi R^{2}\lambda {\left(\frac{\partial T}{\partial r}\right)}_{r=R}\right)-\left(4\pi R^{2}P^{B}\frac{\mathrm{dR}}{\mathrm{dt}}\right)+\left(\sum_{i=1}^{N_{c}}\left(h_{i}-T^{B}C_{v,i}\right)\frac{dN_{i}^{B}}{\mathrm{dt}}\right)\right]$$

(11) $$C_{v,mix}^{*}=\sum_{i=1}^{N_{c}}\left(C_{v,i}-\Psi_{i}\right)N_{i}^{B}$$

(12) $$\Psi_{i}=k\sum_{j=1}^{N_{\theta }}\delta_{i,j}\frac{\left\{{\left(exp\left(\delta_{i,j}\right)\right)}^{2}\left(2\delta_{i,j}-\delta_{i,j}^{2}\right)-\left(\delta_{i,j}^{2}+2\delta_{i,j}\right)exp\left(\delta_{i,j}\right)\right\}}{{\left(exp\left(\delta_{i,j}\right)-1\right)}^{3}};\delta_{i,j}=\frac{\theta_{i,j}}{T^{B}}$$

In the present study, the pseudo steady state assumption implicitly made by Toegel et. al. (2000) [20], for calculating the mixture specific heat was relaxed. A detailed assessment of the impact of the relaxation could not be made due to lack of data relating to collapse pressures and temperatures. Simulations using the present model with and without the relaxation, revealed a variation of the order of 10 – 20% in the predictions for the collapse temperature and pressures. The detailed derivation for equation for $C_{v,mix}^{*}$ is given in the Section A3 of the Supporting Information. The enthalpy of the diffusing species was calculated assuming the bubble interface to be in thermal equilibrium with the liquid. The heat conduction term was calculated analogous to the mass diffusion term described in Section 2.3., by considering the gradient term at the interface with the width of the diffusion layer given according to Equation (14). The rest of the quantities required for the closure of the heat balance equation were calculated using the molecular theory of gases and are given in Section A4 of the Supporting Information.

(13) $${\left(\frac{\partial T}{\partial r}\right)}_{r=R}=\frac{T^{\infty }-T^{B}}{l_{\mathrm{th}}}$$

(14) $$l_{\mathrm{th}}=min\left(\sqrt{\frac{R\kappa }{s}},\frac{R}{\pi }\right)$$

### 2.5 Hydroxyl radicals

The extreme conditions generated inside the bubble at collapse, lead to the production of hydroxyl radicals from the water molecules present in the bubble at collapse. Numerous studies have coupled detailed chemical kinetics models with the equations for mass transfer, heat transfer and the single bubble dynamics, to estimate the rate of generation of radical species at bubble collapse as a function of various operating parameters [7], [20], [26], [27]. However, there is some uncertainty regarding the validity of the kinetics parameter values for the range of extreme conditions generated at bubble collapse. The kinetic parameter values typically estimated over a very limited range of reaction conditions were argued to be the best available resource in absence of reliable kinetics’ data pertaining to the range of conditions applicable for cavitation [7]. Alternatively, the minimization of Gibbs free energy (MGFE) method may be used to estimate the equilibrium compositions of the different radical species based on predictions of the number of water molecules, temperature and pressure conditions at collapse [22], [23], [28], [29].

The minimization of free energy is often used to determine the equilibrium chemical composition in complex systems especially subjected to extreme conditions such as for rocket propulsion [41], gasification [42] and various multiphase separations [43]. Among the choice for the free energy, when the temperature and pressure data is available, the Gibbs free energy is easily minimized while the Helmholtz free energy is easily minimized if temperature and volume are known [41]. The present solver enables the prediction of collapse temperature and pressure and hence the choice Gibb’s free energy for minimization was natural. Considering that the validity of the kinetics model is questionable and the extreme conditions at collapse may promote faster reaction rates thus removing kinetics limitations, the MGFE method may be used to obtain a meaningful upper bound on the rate of hydroxyl radical production. Hence in the present study, the MGFE method was applied to estimate the production of •OH radicals as a function of various operating parameters. The comparison of the MGFE method and a detailed chemical kinetics model was discussed again in more detail with the context of comparison of simulation results in Section 3.2. The Ideal Gas EoS was used to calculate the related thermodynamic properties.

As per the MGFE approach, the composition at equilibrium of all the possible species was calculated by the minimizing the total Gibb’s Free Energy based on the user supplied pressure, temperature and the initial bubble composition. The FactSage Equilib tool [44], available freely under an academic license was used for performing the calculations. The academic license for FactSage uses the original FACTPS library for the properties of pure substances but limits the calculation of the number of elements to H, N and O, which is adequate to simulate the cavitation of water in an environment of air. The pressure, temperature and the composition of the bubble at collapse were supplied to the EQUILIB tool to calculate the number of •OH molecules generated per cavitation event, termed here as the •OH generation rate.

### 2.6 Physical effects due to water jet

It was reported that upon bubble collapse in an unbounded liquid (away from a wall), the intense energy generated in the collapse region is dissipated into the liquid in the form of a spherical shock wave [30]. As the bubble collapses nearer to a wall or a surface, the collapse is no longer spherically symmetric and often generates multiple lesser intensity shockwaves. A consequence of the non-spherical collapse is also the formation of a water jet (known as a jet hammer), which is a small column of water accelerated along the axis of the bubble perpendicular to the wall and pierces through the bubble to the other side with high velocities as the bubble contracts. Analysis of the erosion patterns of cavitating bubbles suggest that both the generated shockwave and the water jet contribute to the cavitation erosion, with the latter becoming prominent as the bubble approaches a surface [12].

Previous investigations relating to the physical effects of cavitation were carried using laser induced cavitation [12], [32], primarily due to the latter’s ability to generate the singular cavitation events and precisely control their internal energy and location with respect to a surface. The initial bubble energy for laser induced cavitation (LC) is typically defined as the internal energy of the initial vapour filled cavity, or more precisely the work done in creating the initial vapour filled cavity. It was shown by Supponen et. al. (2017) [32] that for LC, as the bubble collapses farther away from a wall, the shockwave energy (calculated using measured pressure signals), approached the initial bubble energy. In LC, as there is no varying pressure signal (as for HC or AC) and in the absence of any heat or mass transfer during the collapse phase, the initial bubble energy may be taken equal to the energy dissipated due to bubble collapse. The previous two assertions imply that the bubble internal energy at collapse is entirely converted in the shockwave energy for a bubble collapsing in an unbounded liquid. The bubble internal energy in general may be written as given in Equation (15) and the internal energy at collapse may simply be obtained through simulations.

(15) $$E^{B}=\sum_{i=1}^{N_{c}}C_{v,i}N_{i}^{B}T^{B}$$

(16) $$E_{C,0}^{B}=E_{S}+E_{J}$$

In the absence of viscous dissipation, sound loss or light emission, the collapse energy, in general is dissipated as a combination of a shockwave and a jet hammer. Hence the corresponding energies may be related to the bubble internal energy at collapse as shown in Equation (16).

The shockwave pressure at the collapse location is very high (~10,000 bar) but falls very rapidly as the shockwave travels away from the bubble [36]. Hence, a bubble collapsing in an unbounded liquid does not contribute to the cavitation erosion as much as the jet hammer generated by a bubble collapsing near a wall. It was also shown by Supponen et. al. (2017) [32] that as the bubble approaches the wall, the shockwave energy decreases to zero. Hence, it can be concluded that the collapse energy would entirely be dissipated as the jet hammer energy for a bubble close to the wall. In other words, the kinetic energy of the liquid which, in an unbounded liquid would typically go into the compression of the bubble, due to non-spherical collapse dissipates itself as the jet hammer energy. The jet hammer energy in general may be written as the kinetic energy of the small liquid column piercing the bubble as shown in Equation (17):

(17) $$E_{J}=\frac{1}{2}\rho_{L}\pi R_{J}^{2}R_{\max}U_{J}^{2}=z_{2}\rho_{L}R_{\max}^{3}U_{J}^{2}$$

It was assumed in this case that the velocity of the jet consists of a cylindrical liquid column which extends for a length corresponding to the maximum bubble radius ($R_{\max}$). Further, it was assumed that the jet radius ($R_{J}$) was also proportional to the maximum bubble radius ($R_{\max}$).

It follows from Equation (17) that the jet velocity increases closer to the rigid surface as $E_{J}$ increases to compensate for the decrease in $E_{S}$. This is supported by an intuitive understanding regarding the reason for cavitation erosion, however, contradicts the findings reported by Supponen et. al. (2017) [32] where the jet velocity was shown to reduce closer to the wall. Conversely, Philipp and Lauterborn (1998) [12] have reported an increase in the jet velocity closer to the wall. Supponen et. al. (2017) [32] also reported an increase between the time interval between the instance when the jet pierces the bubble and the total bubble collapse as the bubble approaches the wall. In other words, as the bubble approaches the rigid surface, the jet pierces the opposite bubble wall much earlier, implying that the jet travels the total bubble distance, $R_{\max}$, in progressively shorter times. Thus, it may be inferred that the jet velocity would increase as the bubble approaches the rigid surface. A possible reason for the counter intuitive decreasing trend observed by Supponen et. al. [32] may be that the jet spreads radially along the bubble surface momentarily, just before the piercing and thus might appear to be slowing down. However, after piercing, it may continue along the original direction with the momentum carried forward by the accelerated column of liquid behind it.

The parameter $z_{2}$ used in Equation (17) may be calculated using previously reported experimental results for laser induced cavitation. Philipp and Lauterborn (1998) [12] reported $U_{J}\approx 150ms^{-1}$ for an initial bubble size of $R_{\max}=1.45\times 10^{-3}m$ for a laser induced cavitation of water under atmospheric pressure at 298.1 K. From these values, $z_{2}$ may be estimated by equating the jet energy to the total initial bubble energy for laser induced cavitation as:

(18) $$E_{0}=\frac{4\pi }{3}R_{\max}^{3}\left(P_{0}-P_{v}\right)=z_{2}\rho_{L}R_{\max}^{3}U_{J}^{2}$$

(19) $$z_{2}=\frac{4\pi \left(P_{0}-P_{v}\right)}{3\rho_{L}U_{J}^{2}}\approx 0.0183$$

Hence, the maximum jet velocity may be estimated by assuming that the collapse energy is entirely converted into the kinetic energy for the jet as given in Equation (20). The impact pressure generated by the liquid jet may typically be approximated by using the jet hammer formula [32] as given in Equation (21). It should be noted that the accurate prediction of the water hammer pressure requires the detailed solution of a spherically travelling shockwave equation. The formula for water hammer pressure depends upon both the jet and impact mediums [45]. Equation (21) was used to estimate jet hammer pressure at a point during asymmetric bubble collapse, where the jet pierces the bubble surface. In such a scenario, as both mediums consist of the same liquid, the factor of 0.5 appeared in Equation (21) [45]. Depending on the application scenario, the factor may vary in the range from 0 to 1. For instance, for biological/organic materials the factor would be closer to zero due to lower densities. For applications pertaining to cellular biology or droplet breakage, the factor would be closer to that for water (0.5) as the compositions are like water. For impact with metal surfaces, the factor would be closer to one.

In the present study, the factor of 0.5 was chosen to obtain general guidelines for process design which may be applicable over a broad range of applications. Further, as Equation (21) is linear, the predictions may be modified appropriately. The maximum jet hammer pressure corresponding to the maximum jet velocity (nearest to the wall) may then written be as given in Equation (22).

(20) $$U_{J,max}=\sqrt{\frac{E_{C,0}^{B}}{z_{2}\rho_{L}R_{\max}^{3}}}$$

(21) $$P_{J}=\frac{1}{2}\rho_{L}c_{L}U_{J}$$

(22) $$P_{J,max}=\sqrt{\frac{E_{C,0}^{B}\rho_{L}c_{L}^{2}}{4z_{2}R_{\max,0}^{3}}}$$

The jet velocity and water hammer pressure defined here can be used for estimating physical effects of cavitation on say particle or droplet breakage.

### 2.7 Simulation strategy

The previously described set of equations form a system of ordinary differential equations (ODEs) and may be solved numerically using well established tools and methods. In the present study, the system of ODEs was solved using the ODE15s solver of MATLAB, which is a solver for stiff equations. The absolute and relative tolerances for ODE15s were set to 1x10^-5^ after ensuring that the tolerances did not affect the simulation results. The initial conditions provided to the solver were given by Equations (23), (24), (25), (26). The initial number of molecules in the bubble as per the user specified initial conditions was calculated using the VdW EoS. The VdW EoS, with the total number of molecules as the unknown, turns out to be a cubic equation as given by Equation (30). Equation (30) was solved using the MATLAB root finding function FMINSEARCH with the guess value calculated by solving a linear approximation to the VdW EoS which may be obtained by neglecting the pressure correction factor in the original VdW EoS. Such a guess value ensures the conversion of the root finding algorithm as the solution in not substantially different than the guess value, which is anticipated at ordinary temperatures and pressures.

(23) $${R=R}_{0}$$

(24) $$\frac{\mathrm{dR}}{\mathrm{dt}}=s=0$$

(25) $$N_{i,0}^{B}=x_{i,0}^{B}N_{t,0}^{B}$$

(26) $$T_{0}^{B}=T^{\infty }$$

(27) $$P_{0}^{B}=P^{\infty }+\frac{2\sigma_{L}}{R_{0}}$$

(28) $$V_{0}^{B}=\frac{4\pi R_{0}^{3}}{3}$$

(29) $$N_{t,0}^{B}kT_{0}^{B}=\left(P_{0}^{B}-a\frac{{\left(N_{t,0}^{B}/N_{A}\right)}^{2}}{{\left(V_{0}^{B}\right)}^{2}}\right)\left(V_{0}^{B}-\left(N_{t,0}^{B}/N_{A}\right)b\right)$$

(30) $$P_{0}^{B}V_{0}^{B}-\left(bP_{0}^{B}+kN_{A}T_{0}^{B}\right)\left(N_{t,0}^{B}/N_{A}\right)-a\frac{{\left(N_{t,0}^{B}/N_{A}\right)}^{2}}{V_{0}^{B}}+ab\frac{{\left(N_{t,0}^{B}/N_{A}\right)}^{3}}{{\left(V_{0}^{B}\right)}^{2}}=0$$

A few additional auxiliary equations are also required for the solution of the model. The Antoine type equation used for estimating vapour pressure of water is shown in Equation (31), which was a good continuous function approximation over the investigated range of operation [46]. As done by previous studies [2], [3], [4], the driving pressure was approximated using a sine wave function and is given by Equation (32), (33). This completes the set of equations required for the solution of the model.

(31) $$P_{w}^{v}=133.32exp\left(20.386-\frac{5132}{T^{\infty }}\right)$$

(32) $$P_{L}^{\infty }\left(t\right)=P^{\infty }\left(1-P_{A}^{\text{'}}\sin\left(\omega t\right)\right)$$

(33) $$P_{A}^{\text{'}}=\frac{P_{A}}{P^{\infty }};\omega =2\pi f$$

The MATLAB code for the model is freely available under the GNU license on GITHUB [47]. For a quick reference, a comparison between the various features of the different models is given in Table 1.

**Table 1:** Comparison between the features as reported for various models (VdW: Van der Waals, IG: Ideal Gas, SRK: Sauve-Redlich-Kwong, MGFE: Minimization of Gibbs Free Energy, CKM: Chemical Kinetics Model, Partial: Model contains certain simplifying assumptions).

Reference	EoS	OH Radicals	VdW Pressure Correction	VdW Volume Correction	Radial Variation	Diffusion of Water	Heat Balance	Transient effects ($C_{v,mix}$)
Present	IG/VdW	MGFE	Yes	Yes	No	Yes	Yes	Yes
[7]	SRK	CKM	N.A.	N.A.	Yes	Yes	Yes	Yes
[20]	VdW	CKM	No	Yes	No	Yes	Yes	No
[21]	IG/VdW	CKM	Yes	Yes	No	No	Partial	No
[22]	VdW	MGFE	No	No	No	Yes	Yes	No
[23]	VdW	CKM	No	No	No	Yes	Yes	No
[26]	VdW	CKM	Yes	Yes	No	Yes	Yes	Unclear

### 2.8 Selecting parameter ranges for simulations

The key operating parameters influencing simulation results are – ambient pressure $\left(P_{\infty }\right)$, ambient temperature $\left(T_{\infty }\right)$, the pressure amplitude ratio $\left(P_{A}^{\text{'}}\right)$, initial cavity radius $\left(R_{0}\right)$ and the driving frequency (f). In the present section, the relevant parameter ranges for the values of the various key operating parameters are discussed and identified for further simulation studies. The model reported in the present study is valid for a single bubble contained within a fluid with an oscillating pressure field. Both HC and AC may be suitably described using this mechanistic description and hence the present model may be used to simulate both AC and HC. Indeed, the model may also be used to simulate optical cavitation results, with suitable changes to the initial conditions and the pressure field, although that was beyond the scope of the present study. For laser induced cavitation, the pressure field would be constant, and the initial conditions would need to be calculated accordingly. For instance, the initial size of the bubble would need to be calculated from the laser input power, time of the pulse and the known vapor pressure as described by Supponen et. al. (2017) [32].

HC occurs because the fluid pressure falls to very low values (a small multiple of $P_{V}$) in response to either flow through small constrictions or strong swirling, and such that the pressure fluctuations due to turbulence are sufficient (described later in Section 3.1) to induce cavitation. Further, preliminary simulations and previous studies [7], [20], [21], [22], [23] have reported times scales of the order of a few tens of microseconds (~50 µs) for the complete cycle of the expansion and collapse of a bubble. For typical HC operation with throat or swirl velocities ~ 20 m/s, it may thus be inferred that a cavitating bubble does not travel significantly (~1–2 mm) before collapsing. In various HC devices, the pressure at the throat of the orifice (location of cavitation events) after the onset of cavitation is usually of the order of $P_{V}$ (or a few small multiples thereof). From this point, the pressure recovers gradually (linearly for a standard converging–diverging section) downstream of the throat, until it reaches the ambient pressure/line pressure. The length of this pressure recovery zone for conventional HC devices is usually significantly higher than the total distance that a cavitation bubble would travel. Hence, assuming the mean pressure experienced by the collapsing bubble during its lifespan is constant, and equal to the throat pressure – which is a small multiple of $P_{V}$, is a good approximation.

A key distinction between AC and HC is that for HC, $P_{\infty }$ as experienced by the bubble is the order of $P_{V}$ (or a small multiple thereof), whereas for AC, it is typically atmospheric pressure. In the present study, firstly the influence of ambient pressure, $P_{\infty }$ was investigated wherein the values for $P_{\infty }$ (represented as multiples of $P_{V}$) between ${4P}_{V}$ to $60P_{V}$ were considered. Additionally, due to the key distinction between the HC and AC, the sensitivity with respect to the other parameters was investigated separately for HC ($P_{\infty }={4P}_{V}$) and AC ($P_{\infty }={30P}_{V}$, ~1 atm). The ambient temperature affects cavitation due to the dependence of $P_{V}$ on $T_{\infty }$. Usually cavitation (in both HC and AC) is carried out at near room temperature conditions. Previous studies have investigated cavitation for $T_{\infty }$ values between 290 K and 330 K and hence, was also the range considered in the present study.

There is a lot of uncertainty in specifying $P_{A}^{\text{'}}$ due to difficulties associated with accurate measurement. For sub cavitating regimes in AC, the excitation of the probe surface (or the power input) can be correlated to the pressure amplitude [48]. However, after the onset of cavitation, the emergence of cavitation bubbles is known to cause a steep attenuation of the pressure wave [48], [49], [50]. On increasing the power input to the transducer, the amount of cavitation bubbles also increases, which in turn increases the attenuation of the pressure signal. Thus, the pressure amplitude applied at the source of the transducer quickly dissipates over very short distances (<1cm) thereby making the accurate in-situ measurement of $P_{A}^{\text{'}}$ difficult [48]. Simulation studies dealing with AC have shown that the pressure attenuation due to cavitation bubbles cause nearly a ten-fold decrease in $P_{A}^{\text{'}}$ as opposed to that in theoretical non-cavitating liquids [49]. Nevertheless, simulations also suggest that for AC, $P_{A}^{\text{'}}$ values at least up to 3 may be expected [50]. For HC, computational fluid dynamics (CFD) simulations of devices such as the vortex diode and the orifice have been performed; however, they do not account for the attenuation due to cavitation bubbles [28], [51], [52]. Considering that HC occurs at very low ambient pressures (small multiples of $P_{V}$), pressure time trajectories obtained by tracking multiple single cavities using the discrete particle model coupled with CFD simulations, suggest that like AC $P_{A}^{\text{'}}$ values up to 3 may theoretically be expected [51], [52]. Thus, simulations were performed covering the entire range of values for $P_{A}^{\text{'}}=0\;to\;3$.

Like $P_{A}^{\text{'}}$, there is also a lot of uncertainty in characterising the initial cavity radius, $R_{0}$, due to the small time (~µs) and length (~µm) scales involved. This is especially true for AC or HC, wherein unlike sonoluminescence or laser induced cavitation studies, cavitation bubbles occur in swarms with a range of sizes. Following previous experimental and simulation studies dealing with AC and HC, which have reported bubble sizes in the order of 1–100 µ*m* [7], [20], [28], [53], in the present study the range of $R_{0}=2-50\mu m$ was considered.

The driving frequency (f) in AC is a typically an operating parameter and most of the previous studies typically cover a range between 20 kHz and 200 kHz [7], [20], [21], [22], [23], [28]. In the acoustic emission spectra measured using a hydrophone during AC, peaks were observed at the fundamental frequency (corresponding to the driving frequency) and multiples of this frequencies (which were termed as the ‘harmonics’) [54]. This was argued to be due to the interaction of the cavitation bubbles with the pressure field and suggests that individual bubbles experience frequencies as given by the harmonics and not just the driving frequency. For HC, the driving frequency may not explicitly be controlled and is itself a function of various operating parameters. In such a case, the driving frequency must be inferred from either simulations or measurements. Discrete particle modeling coupled with CFD simulations enables the tracking of the pressure profiles for a few representative cavities which suggest driving frequencies ~ 10 kHz [51], [52]. A simple analysis based on estimating the frequencies of turbulent fluctuations reported by Pawar et. al. (2017) suggests driving frequencies ~ 5 kHz [55]. Like AC, it is expected that cavitating bubbles will experience the driving frequency and the harmonics. Hence, in the present study, the frequencies 5, 10, 20, 40 and 80 kHz were investigated.

It was desirable to investigate the effect of the key parameters on the cavitation performance in terms of the •OH generation and the jet hammer potential. The •OH generation depends mainly on the conditions of the pressure, temperature, and the number of water molecules at collapse. A detailed analysis to estimate the •OH generation by considering the number of water molecules, pressure and temperature at collapse conditions was carried out in the following section. The jet hammer pressure as derived in Section 2.6 was considered. The simulation conditions for the sensitivity analysis are summarized in Table 2 with the base case conditions highlighted. Parameter values for the base case were used for the simulations unless otherwise specified.

**Table 2:** List of parameters for sensitivity analysis ($P_{V}$ was evaluated at 300 K).

Parameter	Values
$P_{\infty }$	${4P}_{V}\left(HC\right),{15P}_{V},1atm\left(AC\right),60P_{V}$
$T_{\infty }$	290, **300**, 310, 320 K
$P_{A}^{\text{'}}$	0.5 to 3; **1.2** (BC)
$R_{0}$	2, **5**, 10, 20, 50 µ*m*
f	10**, 20**, 40, 80 kHz (AC)5, 10, 20, 40 kHz (HC)

The Blake threshold pressure for the onset of cavitation, which is the value of pressure amplitude for a given $P_{\infty }$ and $R_{0}$ above which the bubble is unstable to small disturbances was discussed in Section 3.1 and is given by the Equation (34). The Blake threshold pressure was also plotted for all the conditions considered in the sensitivity analysis.

**Fig. 1:**
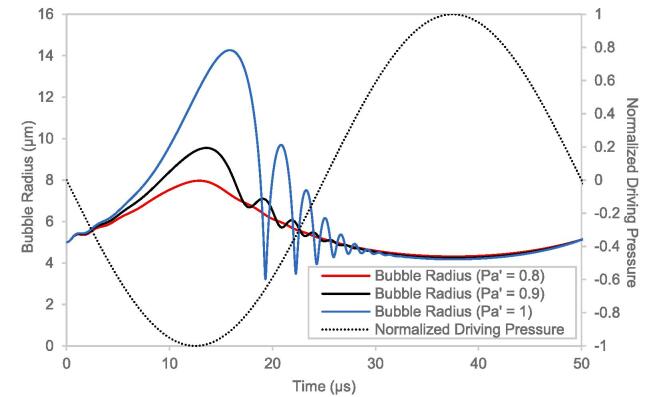
Comparison between the bubble radius profiles representing the transition from non-cavitating to cavitating conditions.

It was observed that when simulations were performed spanning multiple oscillation cycles of the driving pressure, chaotic behavior (a well-defined mathematical concept) emerged at higher values of $P_{A}^{\text{'}}$. At higher values of $P_{A}^{\text{'}}$, the bubble expansion phase is extended which leads to the bubble collapse and subsequent bouncing phases to extend beyond the duration of one cycle. The chaotic behavior was found to emerge when the bubble ‘bouncing’ after the first contraction (as seen in Fig. 1 for $P_{A}^{\text{'}}=1$) interfered with the expansion during the next cycle. The chaotic behavior of single bubble dynamics has been reported previously [56] and was elaborated upon in Section A7 of the Supporting Information as detailed analysis of the chaotic behavior was not the goal of the present study. The analysis of the chaotic behavior of the single bubble dynamics seems pertinent from a mathematical perspective. However, from a practical standpoint and as reported in few previous studies [23], [28], [29], it is useful to consider the bubble dynamics up to the first collapse. Hence, in the present study, influence of the parameters discussed in this section on key performance characteristics (hydroxyl radicals, hammer pressure etc.) was examined by simulating bubble dynamics up to the first collapse.

## 3 Results and discussions

The model was first verified by comparing the present model predictions to previously reported studies. The results of the model verification are presented in Section A5 of the Supporting Information with the key conclusions summarized here. There was an excellent agreement when the present model simulations were compared against simulations reported by Storey et. al. (2000) [7] and Toegel et. al. (2000) [20] for the bubble radius and number of water molecules’ profiles. The model was also verified by comparison against previously published literature (both simulated and experimental) in terms of the percentage of water molecules contained within the bubble at collapse as shown in Table 3. Simulations revealed that there is no significant effect of the choice of EoS on the simulation results for the bubble radius and number of water molecules’ profiles as described in Section A5 in the Supporting Information and was also reported by Kerboua et. al. (2017) [21].

**Table 3:** Comparison between predictions using different models for the percentage number of water molecules in the bubble at collapse (VdW: Van der Waals, EOS: Equation of State).

Case	R0 (*µm*)	Pa (bar)	T (K)	f (Hz)	Moss et. al. [36]	Storey et. al. [7]	Toegel et. al. [20]	VdW EOS	Ideal Gas EOS
A1	6	1.4	293.15	20.6	40	33	33	41	41
B1	4	1.32	293.15	20.6	36	27	23	31	31
C1	2.1	1.29	293.15	20.6	30	22	14	21	21

For the collapse temperature, comparison with results reported by Storey et. al. (2000) [7] enabled the verification of the key time scales and the confirmation of the order of magnitude of the temperature rise. An exact agreement, as observed in the bubble radius profile was not expected due to differences in assumptions concerning the bubble internal mixing conditions in both the models. The pressure and temperature profiles around the time of bubble collapse, were not substantially affected by the choice of the EoS as shown in Figure in Section A5 of the Supporting Information. A good agreement was observed with the previous models for the water content at collapse under various conditions. As seen previously, there was no effect of the choice of EoS on the water content at collapse. The values for collapse pressure have not been reported and hence a comparison could not be made. The model was thus sufficiently verified and was used further to investigate different aspects related to cavitation as described in the subsequent sections.

### 3.1 Onset of cavitation

Simulations were first carried out to investigate the onset of cavitation. It is generally regarded that the cavitation occurs during the pressure recovery phase (for HC) or the pressure increase phase (in AC) after the initial pressure decrease phase. In the present study, it was observed through simulations that, although an increase in pressure is responsible for the cavitation, the point of collapse does not in general coincide with the point of complete pressure recovery (ambient pressure). Mathematically, cavitation may be interpreted as a sharp and significant (order of magnitude) increase in the maximum pressure and temperature. At lower values of amplitude of fluctuating pressure ratio, $P_{A}^{\text{'}}$ for which no cavitation occurred, the bubble expanded and contracted in sync with the driving pressure signal and no perturbations were observed as shown in Fig. 1 $\left(P_{A}^{\text{'}}=0.8\right)$. As $P_{A}^{\text{'}}$ was increased, at a value slightly below the cavitation threshold $\left(P_{A}^{\text{'}}=0.9\right)$ perturbations were observed during the contraction phase of the bubble. However, there was no cavitation observed corresponding to these perturbations. The cause of these perturbations may be cursorily attributed to a sudden switch from bubble expansion to contraction. As $P_{A}^{\text{'}}$ was increased to above the cavitation threshold $\left(P_{A}^{\text{'}}=1\right)$, the perturbations developed into significant contractions with significantly high pressure and temperatures recorded at the corresponding instances which indicated cavitation. It should be noted that the radial trajectory plot shown in Fig. 1 superimposed on the normalized pressure is representative of all cavitation events (HC and AC) considered in the present study. Naturally, the onset would occur at different points depending upon the prevalent conditions but the overall physics behind the onset of cavitation as described in this section would be retained.

A detailed analysis dealing with the inception of cavitation by analyzing the contribution of the various terms in the bubble dynamics equation is presented in Section A6 of the Supporting Information. It was observed that above the cavitation threshold, the bubble contraction is accelerated due a compounding effect of the increasing bubble wall velocity contributing to increasing the acceleration. The compounding effect thus leads to a violent collapse of the bubble or cavitation. This is reminiscent of the Blake threshold pressure given by Equation (34), which is an elegantly derived formula for the pressure amplitude above which the expansion or contraction of the bubble becomes unstable [57]. The analysis for the Blake threshold pressure is purely based on surface tension considerations.

(34) $$P_{B}^{*}=P_{\infty }-P_{v}+\sqrt{\frac{32\sigma^{3}}{27R_{0}^{3}\left(P_{\infty }-P_{v}+2\sigma /R_{0}\right)}}$$

Under typically room temperature and pressure conditions and for a 5 µm initial bubble radius, $P_{B}^{*}$ has a value slightly above the atmospheric pressure. Above the Blake threshold pressure, any small disturbance in terms of bubble radius or the driving pressure, in either direction leads to a corresponding uncontrolled expansion or contraction [57]. Cavitation may thus be thought of as a combination of two factors, the first obvious factor being that the pressure amplitude be above the Blake threshold pressure. The second less obvious factor is that the disturbance required to cause the uncontrolled contraction is supplied in the form of the perturbations of the bubble radius observed (in Fig. 1) during the contraction phase near the cavitation threshold conditions $\left(P_{A}^{\text{'}}=1\right)$. However, as the latter is always expected to occur conditional on the pressure amplitude being above the Blake threshold pressure, the former seems to be a necessary and sufficient criterion for the onset of cavitation. Thus, $P_{B}^{*}$ is the threshold pressure amplitude above which exciting the bubble would lead to cavitation.

### 3.2 Chemical effects – Generation of hydroxyl radicals

**Fig. 2:**
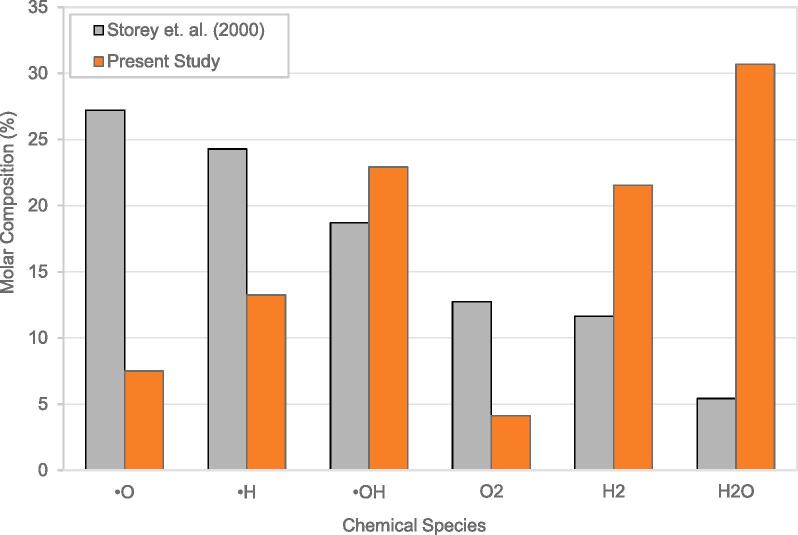
Comparison between simulation results of Storey et. al. (2000) [7] and the present study for the percentage composition of different species at collapse.

Simulated results for the •OH generation for a set of operating conditions were reported previously by Storey et. al. (2000) [7]. It was demonstrated in the Section A5 of the Supporting Information that simulation results of the present solver showed a good agreement with those reported by Storey et. al. (2000) [7] for the bubble radius and the number of water molecules profiles. The collapse temperatures were seen to be under predicted in comparison with those reported by Storey et. al. (2000) [7] at the center of the bubble around the time of collapse but had the same order of magnitude. A lower value was expected as a ‘lumped’ heat transfer model was used in the present study as opposed to one considering radial variation (change in temperature within the bubble along its radius assuming radial symmetry) as done by Storey et. al. (2000) [7]. A comparison between the bubble internal compositions using the present model and those reported by Storey et. al. (2000) [7] under similar operating conditions (described in Section A5, Supporting Information) is shown in Fig. 2. The compositions shown in Fig. 2 were reported excluding Argon as a chemical species which would remain constant throughout the cavitation process. Like the radial and number of water molecules trajectories, the relevant data was extracted using the PlotDigitizer tool and the associated error in the data extraction (corresponding to the line thickness) was negligible in comparison with the considered data. It was observed that there a mismatch between the compositions reported by Storey et. al. (2000) [7] and those predicted using the present model, although the most important species were consistent.

A possible source of mismatch may be attributed to the exclusion of an explicit reaction kinetics model and differences in modeling approaches. Storey et. al. (2000) [7] reported that the chemical kinetic model was not strictly valid under the extreme collapse conditions (high temperature, pressure), however, enabled the best possible estimate. On the other hand, the minimization of Gibb’s free energy (MGFE) method used in the present study, is another popular approach for modeling rapid chemical transformations under extreme conditions and presents an alternative reliable estimate. One valid criticism of the MGFE method is that in the time scales within which the collapse conditions exist, the reactions would not have proceeded to equilibration and that the reaction rates may be over predicted. However, the composition of water molecules trapped in the bubble for Storey et. al. (2000) [7] was significantly depleted in comparison to the present study, indicating significant conversions due to reactions had occurred - which contradicts the supposed over prediction. This further signifies the importance of considering alternative modeling approaches especially given the extreme conditions that are likely to exist during cavitation.

The model was then used to estimate generated hydroxyl radicals. It should be noted that the estimation of hydroxyl radicals, either based on experiments or based on physics based models like the one discussed in this manuscript is fraught with several uncertainties. The number of OH radicals generated per oscillation cycle was determined experimentally by Didenko and Suslick (2002) [58] using sensitive fluorescent analyses. Unfortunately, the confidence intervals on estimated values or potential sources of errors were not discussed by the authors. Under similar conditions the present model overestimates the OH radicals generation rate. It should be noted that the estimation of hydroxyl radicals using cavity dynamics mainly depends on following three aspects:

• Diffusion of species (water as well as dissolved gases like oxygen and nitrogen) in cavity
• Species and reactions (with or without ionic reactions)
• Reaction kinetics or equilibrium constants

In the present study, the diffusion of dissolved gases (such as O_2_ and N_2_) into the bubble was not considered. The inclusion of these in the bubble collapse calculations would lead to the formation of various other chemical species that would compete with the OH radicals (prominently the nitrite ions as described by Didenko and Suslick, 2002 [58]) and may explain the over prediction. Yasui et. al. (2005) [59] included the diffusion of dissolved gases into the cavitation bubble as well as ‘excluded volume effects’ as reported by Toegel et. al. (2002) [60] and obtained a good match with the experimental results for OH radicals reported by Didenko and Suslick (2002) [58]. The OH radical generation rate along-with that of other chemical species of interest was determined by Yasui et. al. (2005) [59] by considering a detailed chemical kinetics model. However, ionic reactions (reported by Didenko and Suslick, 2002 [58]) were not considered in the chemical kinetics model and the applicability of the chemical kinetics model for the extreme conditions obtained for cavitation was not discussed. These considerations serve to favour the generation of other chemical species at the expense of the OH radical generation rate in comparison with the present approach. To the author’s knowledge, presently there are no modelling studies considering ionic reactions to explain the observations reported by Didenko and Suslick (2002) [58]. Indeed, the model may be further refined by the inclusion of diffusion of dissolved gases and the excluded volume effects as done by Yasui et. al. (2005) [59] and by considering ionic reactions. However, considering the uncertainties in estimating diffusion coefficient and other transport properties as well as in estimating reaction kinetics at cavity collapse conditions in absence of reliable estimates of transport properties and reaction kinetics at cavity collapse conditions, the presented model based on equilibrium assumption (MGFE method) provides a useful method to estimate an upper limit of OH radical generation rate. The comparison of estimated percentage of OH radicals using the present model with the predictions of the model of Storey et al. [7] is whon in Fig. 2. It may be noted that the •OH predicted by both the models agreed reasonably well. Based on preceding analysis, the present model was thus used to estimate the upper limit for the •OH generation and influence of various operating parameters on the estimated •OH generation rate.

The MGFE method used in this work requires the input of the collapse event information, namely - collapse pressure, temperature and the number of water molecules, in order to estimate •OH generated per collapse event or the •OH generation rate (OHG). The required collapse event information for each case was first generated through simulations by varying the operating conditions and the key results are presented separately in Section A8 of the Supporting Information. One of the key conclusions was that $P_{B}^{*}$ was shown to be a sufficient criterion for capturing the onset of cavitation. Under certain conditions, the cavitation was seen to initiate for $P_{A}<P_{B}^{*}$, however, the orders of magnitude of the collapse pressures and temperatures were insignificant in comparison for those where $P_{A}>P_{B}^{*}$.

**Fig. 3:**
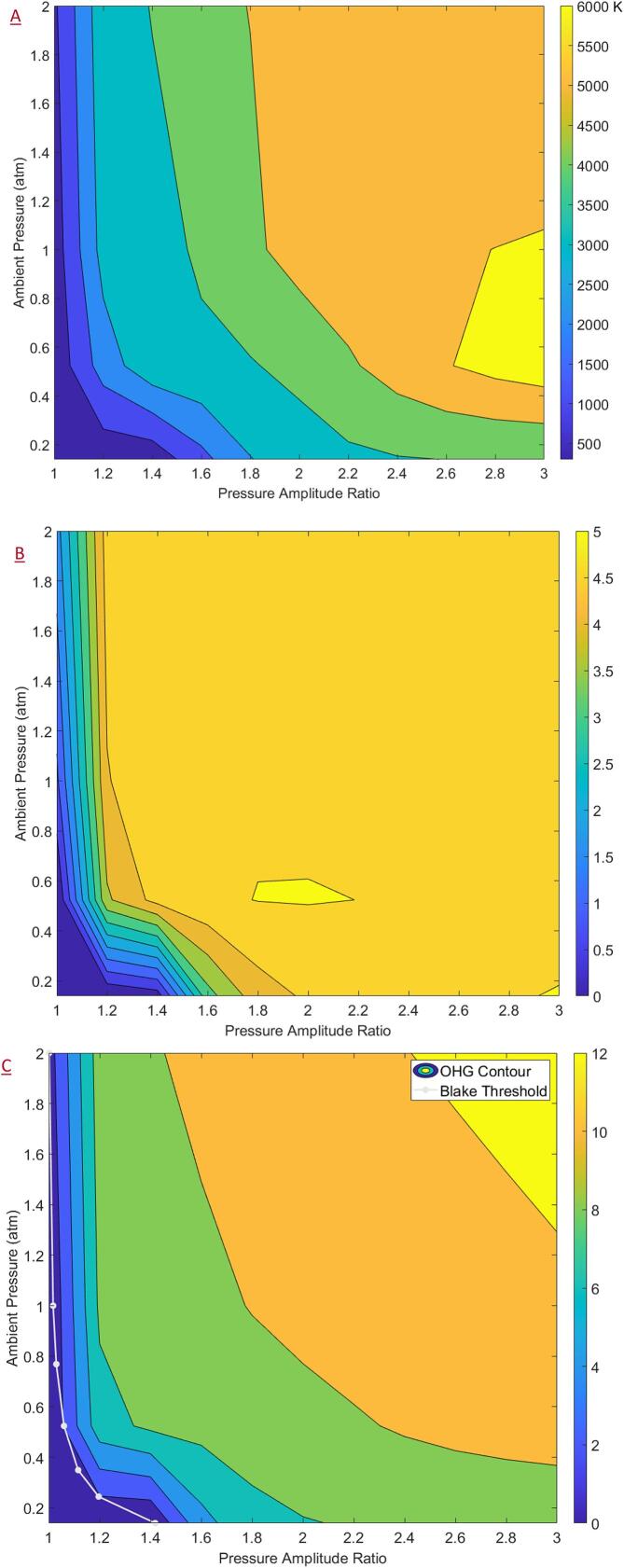
Contour plots as a function of the pressure amplitude ratio and the ambient pressure of (a) collapse temperatures (b) logarithm of collapse pressures and (c) the logarithm of •OH generation plotted with Blake threshold.

The contour plots were generated using the MATLAB 2020b function ‘CONTOURF’ which constructs a contour map from a discrete set of target variable values obtained along a grid through simulations. The contours were plotted using distinct colour bands make it easier to discern various orders of magnitudes. Smoother contours may be obtained by performing more simulations or as is typically done – by interpolating between the known values. However, considering the wide range of uncertainties associated with the estimated parameters, distinct bands were used to identify possible order of magnitude improvements in the process. A continuous colour bar was shown to indicate a continuous range of values obtained through simulations. Contour plots generated for the collapse temperatures and (logarithm of) collapse pressures for the case of varying ambient pressures are shown in Fig. 3A and 3B respectively. Collapse temperatures and pressures of the order of ~ 5,000 K and ~ 10,000 bar were respectively observed, confirming previously reported estimates.

Since effective ambient pressure for AC and HC is different, influence of the ambient pressure and $P_{A}^{\text{'}}$ on •OH generation was first examined as shown in Fig. 3C. OHG (plotted as the logarithm) as high as 10^12 •OH per collapse event may be obtained under high ambient pressures and pressure amplitude ratios. The OHG was found to be sensitive to the ambient pressures more so at lower pressure values, and hence the influence of the rest of the parameters was studied separately for AC ($P_{A}=1bar$) and HC ($P_{A}=4P_{v}\;0.15bar$) using the respective ambient pressures. Increasing the ambient pressure was seen to increase the OHG across all $P_{A}^{\text{'}}$ values and similarly, increasing $P_{A}^{\text{'}}$ was seen to increase the OHG for all ambient pressures. Increasing the ambient pressure enabled the earlier onset of cavitation with respect to $P_{A}^{\text{'}}$. It should be noted that the diffusion of the •OH into the bulk liquid and the time scales for the recombination reaction (•OH and •H) in competition with the other reactions of interest would need to be considered to obtain meaningful quantitative estimates. The presented contour plots serve well to gauge the effect of various operating variables and meaningfully guide process design.

#### 3.2.1 •OH generation via acoustic cavitation

**Fig. 4:**
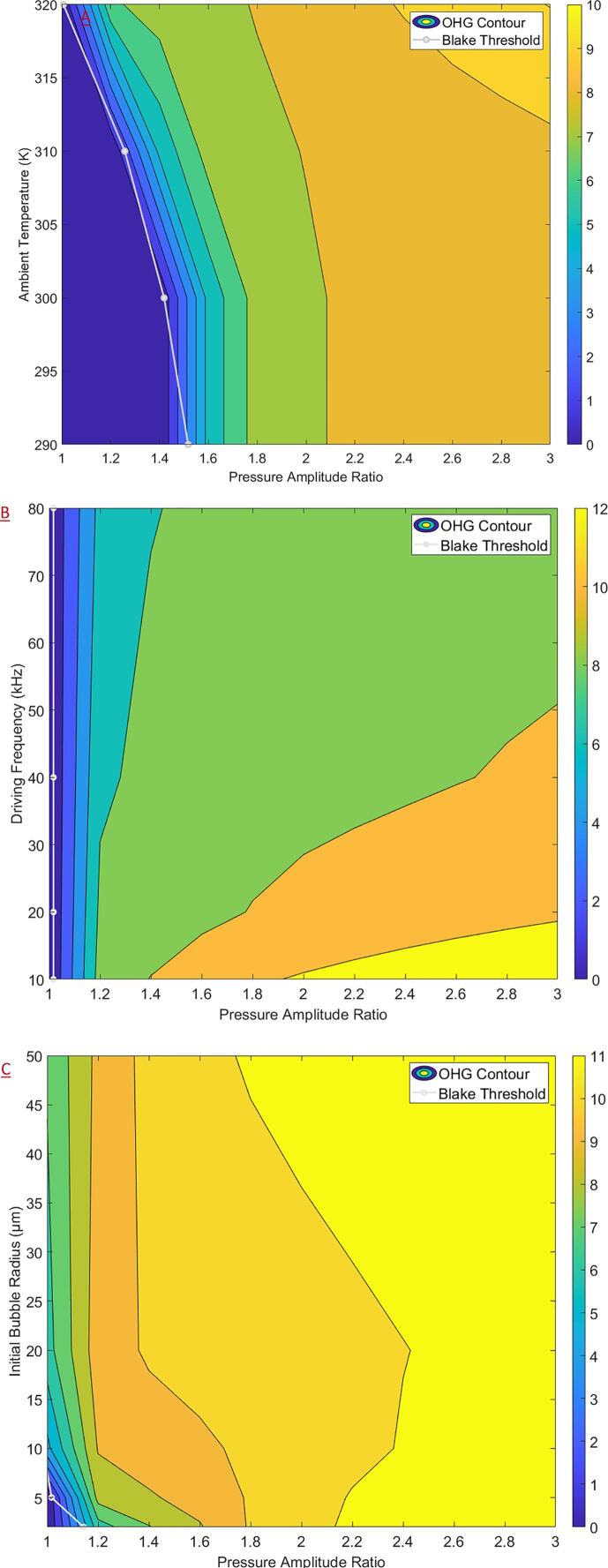
Contour plots (plotted with Blake threshold) of the logarithm of •OH generation for acoustic cavitation as a function of the pressure amplitude ratio and (a) ambient temperature (b) driving frequency and (c) initial bubble radius.

The contour plots of the logarithm of OHG with respect to the $P_{A}^{\text{'}}$ as the primary variable, and the ambient temperature, driving frequency and the initial bubble radius each as the secondary variables are shown in Fig. 4. For all cases considered, it was found that the OHG increased with increasing $P_{A}^{\text{'}}$, and an OHG of the order 10^10 •OH per collapse event may be obtained over most of the operating space. It was found that increasing ambient temperatures were seen to enhance the OHG at intermediate values of $P_{A}^{\text{'}}$. Decreasing the driving frequency increased the OHG, but the effect was most pronounced only at higher $P_{A}^{\text{'}}$. Increasing the initial bubble radius was seen to increase the OHG but only at lower values of $P_{A}^{\text{'}}$ and was seen to enable the earlier onset of cavitation with respect to $P_{A}^{\text{'}}$. For all plots, it may be observed that the Blake threshold pressure may be used as a criterion for predicting the onset of ‘useful cavitation’ for given process conditions.

#### 3.2.2 •OH generation via hydrodynamic cavitation

**Fig. 5:**
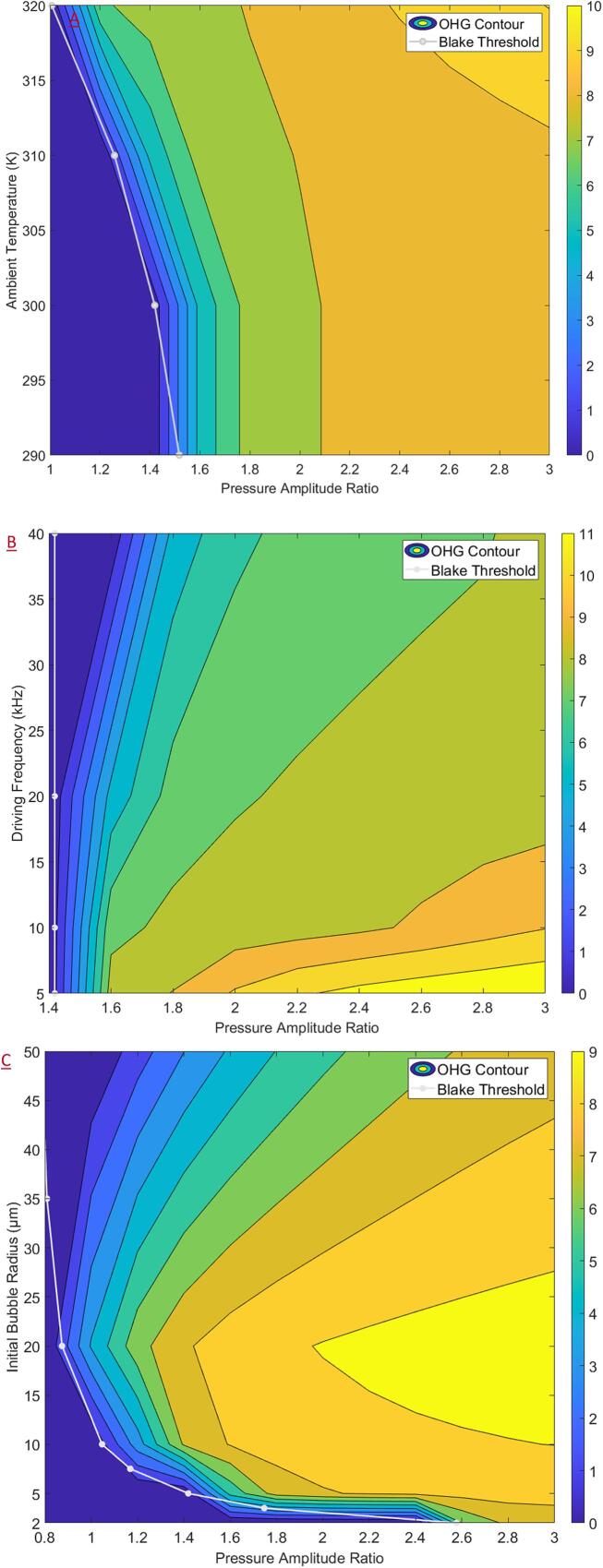
Contour plots (plotted with Blake threshold) of the logarithm of •OH generation for hydrodynamic cavitation as a function of the pressure amplitude ratio and (a) ambient temperature (b) driving frequency and (c) initial bubble radius.

The contour plots of the logarithm of OHG considering $P_{A}^{\text{'}}$ as the primary variable and the ambient temperature, driving frequency and initial bubble radius as the secondary variable, for HC is shown are Fig. 5. Overall, for HC an OHG of the order 10^7 to 10^8 •OH per collapse event may be obtained covering much of the operating space which was found to be roughly two orders of magnitude lower than AC. As was the case for AC, the Blake threshold pressure was found to provide a good estimate for the onset of ‘useful cavitation’ for HC. For all the cases considered, increasing $P_{A}^{\text{'}}$ was found to increase the OHG. For the case of HC, it is difficult to explicitly determine $P_{A}^{\text{'}}$, however, it is a function of the local turbulence characteristics. Hence, fluid dynamic investigations into the impact of increasing the local turbulent fluctuations (and possibly manipulating fluctuation frequencies) on the cavitation performance seem to be promising.

For the ambient temperature, as shown in Fig. 5A, increasing the temperature was seen to increase the OHG for lower to intermediate values of $P_{A}^{\text{'}}$ and enabled an earlier onset of cavitation with respect to $P_{A}^{\text{'}}$. In a practical scenario, this implies that increasing the temperature may enable an earlier onset of cavitation (at lower flow rates), or an increased OHG for a constant $P_{A}^{\text{'}}$ (described by the local turbulence characteristics) thus potentially improving process efficiency. However, increasing temperatures will also impact reaction rates, dissolved gas concentration and turbulence characteristics which may counteract the positive effect described previously. Hence, the effect of ambient temperature on process performance presents an important and promising avenue for further investigation.

**Fig. 6:**
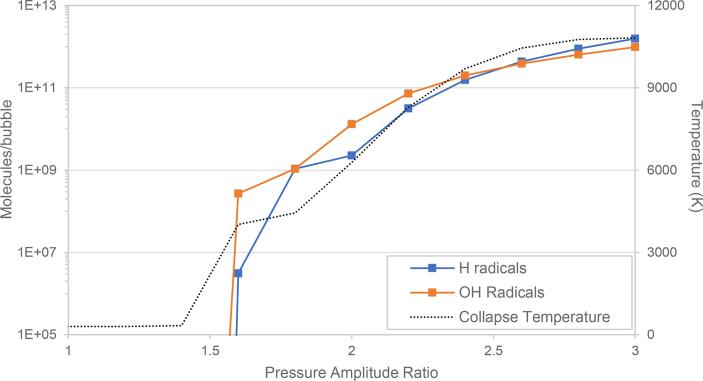
Comparison between the •OH and •H generation, collapse temperature versus $P_{A}^{\text{'}}$ profiles for 5 kHz driving frequency (only observed at high temperatures ~ 10,000 K).

From Fig. 5B it was found that reducing the driving frequency increased the OHG with the effect being most pronounced at higher $P_{A}^{\text{'}}$. At higher driving frequencies for HC, the Blake threshold pressure (which is constant) may not yield a sufficient criterion for useful cavitation. At the lowest frequency − 5 kHz, it was observed that the temperature rose beyond 10,000 K (Figure A5.2 in Supplementary Information) at very high values of $P_{A}^{\text{'}}$, which was correlated to an increase in the •H generation – even surpassing the OHG under those conditions as shown in Fig. 6. In all other instances covered in the present study, the temperature was lower and the generation of •H in comparison with •OH was not significant. In such a scenario, it is expected that after cavitation the generated •OH would recombine with the produced •H effectively decreasing the overall oxidation potential and process efficiency.

In Fig. 5C the contour plot for the logarithm of OHG as a function of the initial bubble radius and $P_{A}^{\text{'}}$ is shown which conveyed some interesting features. The plot indicated an optimal size of 20 µm for the initial bubble radius in terms of increasing the OHG and enabling an earlier onset. The Blake threshold pressure indicated an earlier onset of cavitation with respect to $P_{A}^{\text{'}}$on increase bubble radii, however, this was not found to translate into higher OHG as for the other cases. On referring to the respective contours for collapse pressures and temperature (Figure A8.1 in Section A8.7 of the Supplementary Information), the same qualitative trends were observed. However, for higher bubble radii even though cavitation (defined as the abrupt and substantial increase in the pressure and temperature conditions) was observed, the collapse conditions were comparatively milder (temperature ~ 1,000 and pressure ~ 100 bar) which would explain the reduction in OHG. Thus, deaerating the process fluid (to avoid uncontrolled nucleation of dissolved gases) coupled with the controlled introduction of bubbles before the cavitation zone may lead to significant improvements in process efficiencies and presents another promising avenue for further investigation.

### 3.3 Physical effects - jet hammer pressure and jet velocity

The erosion mechanism due to cavitation is attributed to the water jet which forms due to the non-spherical collapse of a cavity near a surface. The water jet strikes the adjacent surface normally with very high velocities locally leading to high instantaneous pressures at the point of impact, termed as the jet hammer pressure (JHP). The jet hammer pressures generated are well beyond the compressive strength of the material and hence result in material fatigue and eventually failure due to the repeated shocks resulting from recurring local cavitation events. These may be broadly described as the physical effects of cavitation and as mentioned are also beneficial in certain applications. In order to describe these physical effects, new formulae were proposed to estimate the JHP and the water jet velocity (JV) using the single cavity model results proposed in Section 2.6. In this section, effects of various operating parameters on the JHP are discussed.

**Fig. 7:**
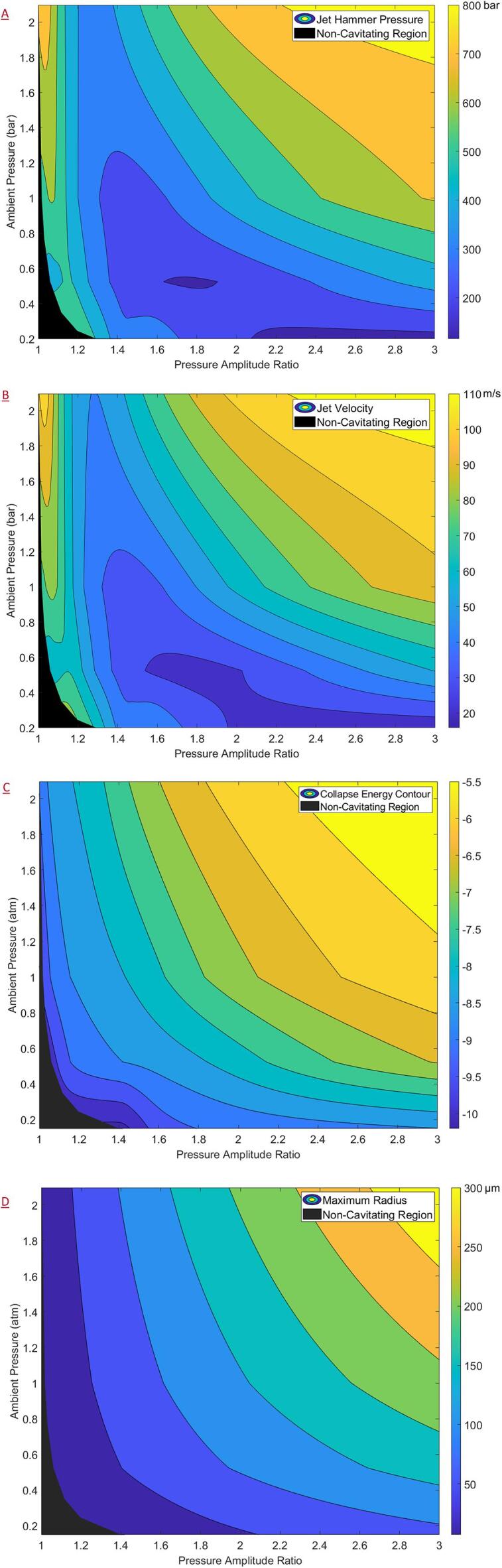
Contour plots (plotted with non-cavitating region) as a function of the pressure amplitude ratio and the ambient pressure of (a) jet hammer pressure (b) jet velocity (c) logarithm of collapse energy (d) maximum bubble radius.

Like the analysis for OHG, the influence of ambient pressure on physical effects was first examined. The contour plots for the relevant key variables of JHP, JV, collapse energy (logarithm) and maximum bubble radius are shown in Fig. 7. It should be noted that the following analysis was only carried out for the region beyond the Blake threshold. Owing to the formulae for the JHP (Equation (22)) and JV (Equations (20)), a numerical value may be obtained even for regions before the Blake threshold. However, these values should be treated merely as numerical artefacts as they do not have a physical interpretation. For this reason, the region below the Blake threshold was blacked out in the following contour plots and denoted simply as the non-cavitating region. However, during contour generation, only the values of JHP which were not required for the obtaining a smooth contour near the threshold were excluded. The contour plots were generated using the MATLAB 2020b function ‘CONTOURF’ which constructs a contour map from a discrete set of target variable values obtained along a grid through simulations.

From Fig. 7A and 7B, it may be observed that the JHP and JV follow similar trends which is expected due to the linear relationship between the two as described by Equation (21). Adjacent to the non-cavitating region, the JHP values were found to be around 500 to 600 bar and the corresponding JV values were found to be around 70 to 80 m/s. Interestingly, for ambient pressures, the JHP was found to go through a minimum value region with respect to $P_{A}^{\text{'}}$ before increasing again for higher values of $P_{A}^{\text{'}}$ which, if validated, would have interesting consequences. The trend for a minimum in the JHP versus $P_{A}^{\text{'}}$ profiles was not discernible from the corresponding collapse energy (logarithm) and maximum radius plots shown in Fig. 7C and 7D respectively. However, owing to the non-linear formula as described by Equation (22) such a trend may be expected. The collapse energy and the maximum bubble radius were found to increase with increasing ambient pressure and $P_{A}^{\text{'}}$ with the collapse energy of the order of a few nano-Joules and the maximum radius around 50 μ*m* adjacent to the non-cavitating region (initial bubble size of 5 μ*m*).

Due to the high sensitivity of the JHP to the ambient pressures, the sensitivity analysis was performed separately for the cases of AC and HC in the subsequent sections as was done for the case of OHG.

#### 3.3.1 Acoustic cavitation:

**Fig. 8:**
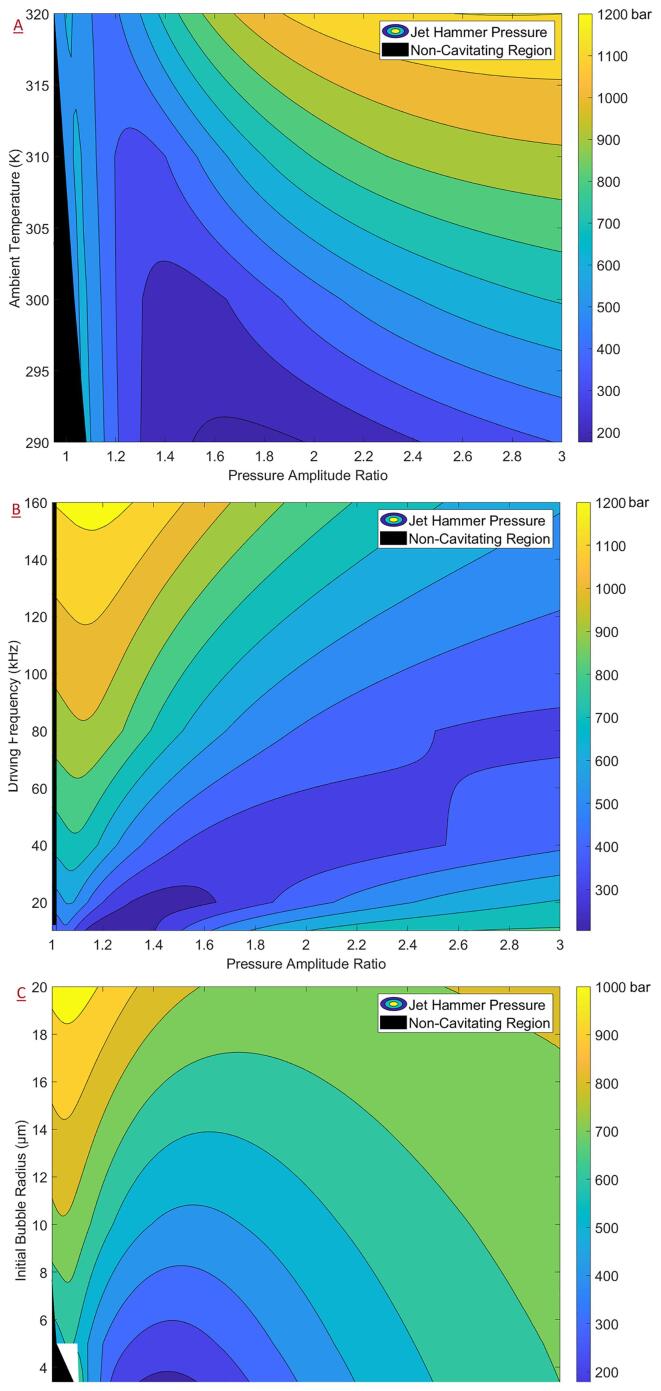
Contour plots (plotted with Blake threshold) of the jet hammer pressure for acoustic cavitation as a function of the pressure amplitude ratio and (a) ambient temperature (b) driving frequency and (c) initial bubble radius.

Following the analysis presented in the previous section, contour plots of JHP for AC are reported in the present section considering $P_{A}^{\text{'}}$ as the primary variable and the ambient temperature, driving frequency and initial bubble radius as the secondary variables. The non-cavitating region is defined as the region having $P_{A}^{\text{'}}$ values less than the corresponding Blake threshold for those conditions. The effect of the ambient temperature (Fig. 8A) was like that of the ambient pressure described previously with JHP values of around 500 bar just adjacent to the non-cavitating region and an area of minimal JHP values for intermediate $P_{A}^{\text{'}}$ values for a given ambient temperature. For intermediate to higher $P_{A}^{\text{'}}$, the JHP increased with increasing temperature. Further, for increasing temperatures, the range of values which fall within the minimal region decreases. Hence, for applications requiring the enhancement of the physical effects of cavitation, it would be recommended to increase temperature and $P_{A}^{\text{'}}$. However, it should also be noted that increasing temperatures may also complicate the process dynamics through other mechanisms. For instance, an increased dissolved gas concentration may lead to cloud cavitation effectively deteriorating cavitation performance. For applications requiring the physical effects to be minimized, it would be recommended to operate at low ambient temperatures. Interestingly, the chemical effects of AC are not severely impacted by decreasing temperatures as described in Section 3.2.1 and hence, the effect of ambient temperature presents a promising area of research.

For the driving frequency (Fig. 8B), adjacent to the non-cavitating region (low values of $P_{A}^{\text{'}}$), the JHP was seen to increase with increasing driving frequencies, reaching values as high as 1200 bar at the highest considered frequency (160 kHz). For intermediate to high values of $P_{A}^{\text{'}}$, the JHP was seen to go through a minimum for increasing driving frequencies with relatively lower values within the range of 300 to 500 bar. In contrast with the case of ambient temperature, at higher driving frequencies, increasing $P_{A}^{\text{'}}$ was seen to reduce JHP demonstrating a strong interdependence of the operating parameters with regards to the physical effects of cavitation.

For the effect of initial bubble radius (Fig. 8C), increasing the bubble size was seen to increase the JHP across all the $P_{A}^{\text{'}}$. The JHP values reached as high as 1000 bar for highest considered initial bubble radius (of 20 μ*m*) adjacent to the non-cavitating region (low values of $P_{A}^{\text{'}}$). The JHP was seen to go through a minimum for increasing values of $P_{A}^{\text{'}}$ at all bubble radii – reinforcing the strong interdependence of operating parameters for the physical effects of cavitation. Due to an issue concerning contour plot generation, the JHP values could not be computed adjacent to the non-cavitating region for low initial bubble radius values (unfilled space within the contour). This issue is discussed in greater detail in the Section 3.3.2.

Typical operating conditions would be expected to be in the region adjacent to the non-cavitating region. Further, increasing $P_{A}^{\text{'}}$ is difficult due to the attenuation effects of cavitation on the pressure signal [50] effectively shifting the value of $P_{A}^{\text{'}}$ towards the lower side. It is worthwhile to note that in contrast to the other operating parameters, the driving frequency may be used to enhance the JHP especially at lower $P_{A}^{\text{'}}$ and seems to be a promising avenue for further research to enhance the physical effects of cavitation.

#### 3.3.2 Hydrodynamic cavitation:

**Fig. 9:**
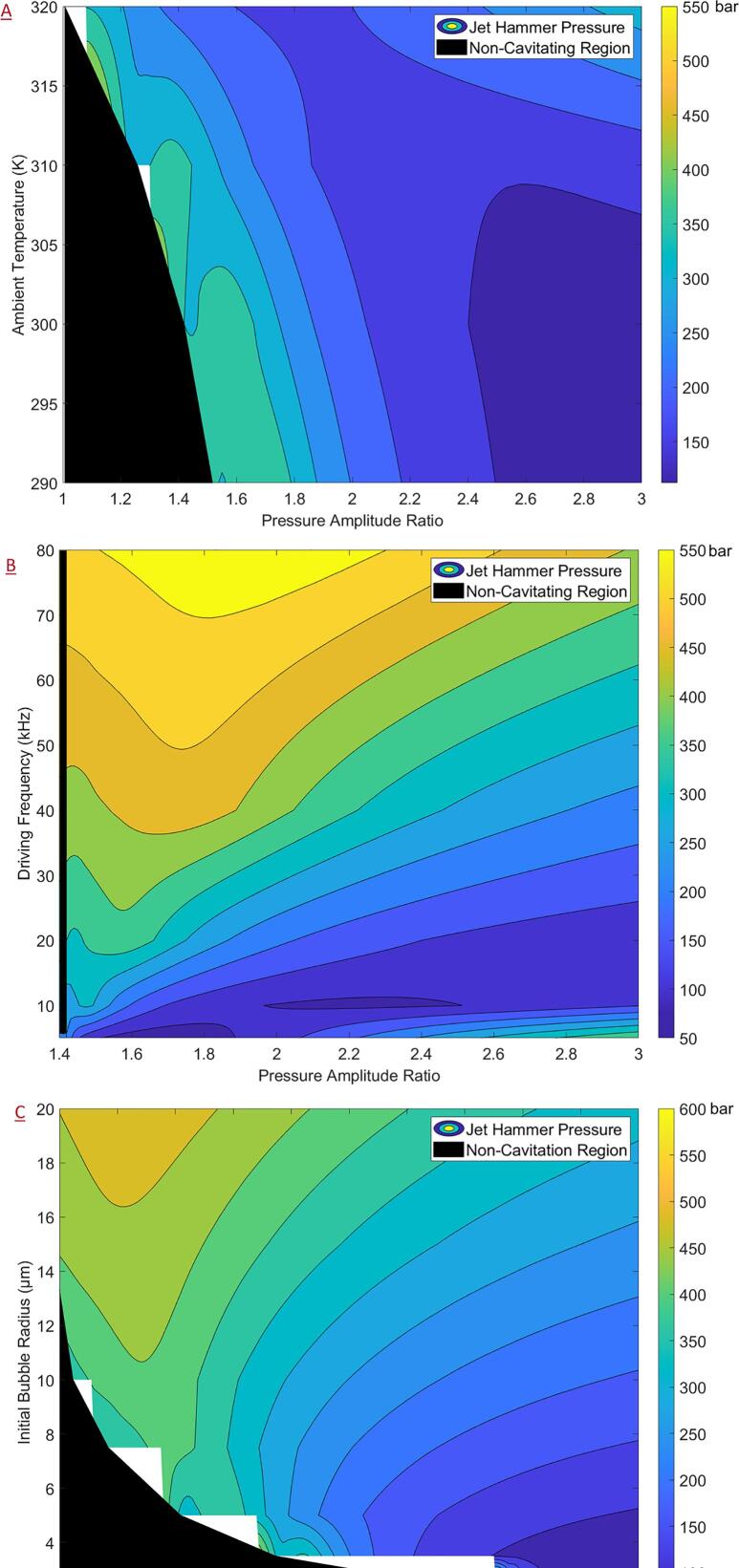
Contour plots (plotted with non-cavitating region) of the jet hammer pressure for hydrodynamic cavitation as a function of the pressure amplitude ratio and (a) ambient temperature (b) driving frequency and (c) initial bubble radius.

Contour plots of the JHP for HC as a function of $P_{A}^{\text{'}}$ as the primary variable and ambient temperature, driving frequency and initial bubble radius as the secondary variables are shown in Fig. 9. The non-cavitating region is defined as the region having $P_{A}^{\text{'}}$ values less than the corresponding Blake threshold for those conditions. Due to an issue concerning contour plot generation, the JHP values could not be computed adjacent to the non-cavitating region under certain circumstances (unfilled space within the contour). This issue was especially pronounced for HC due to the wide variation in the Blake threshold pressure over the parameter space considered and could not be resolved despite significant efforts. The issue arose due to the discrete nature of the contour generation, a steep change in the non-cavitating region slope and the exclusion of JHP values within the non-cavitating region in generating the contour plots. The JHP formula given by Equation (22) does allow calculation for the non-cavitating region. However, the JHP values in the non-cavitating region were excluded as they skewed the contour plots ranges – impacting the result interpretation, without adding any value.

As shown in Fig. 9A, increasing the ambient temperature enabled the early onset of cavitation with respect to $P_{A}^{\text{'}}$ which is important for HC. The JHP adjacent to the non-cavitating region was seen to be around 350 bar although, the results are affected by the contouring issue described earlier. Nevertheless, this presents a promising further line of investigation – particularly for obtaining cavitation at lower flow (increasing the residence times) rates while retaining the physical effects of cavitation. Increasing $P_{A}^{\text{'}}$ was seen to reduce the JHP for most of the range of ambient temperatures considered. As shown in Fig. 9B, increasing the driving frequency was shown to increase the JHP across all $P_{A}^{\text{'}}$ while increasing $P_{A}^{\text{'}}$, for a given driving frequency, was not seen to affect the JHP significantly. For higher values of driving frequency, JHP as high as 550 bar may be obtained. For the initial bubble radius as shown in Fig. 9C, increasing the bubble size was seen to increase the JHP while increasing $P_{A}^{\text{'}}$ caused a reduction in the JHP. Increasing the bubble size also enabled the earlier onset of cavitation which would be useful from a practical point of view. The JHP was seen to be around 400 bar adjacent to the non-cavitating region – immediately after the onset of cavitation.

It should be noted that $P_{A}^{\text{'}}$, driving frequency and the initial bubble radius are not explicitly controlled operating variables but rather a consequence of more tangible operating variables such as the flow rate, pressure, device geometry, dissolved gas content and so on. Specifically, for HC, $P_{A}^{\text{'}}$ and the driving frequency may loosely be considered as functions of the local flow and turbulence characteristics. Hence, the impact of flow and turbulence characteristics on the cavitation performance seems like a promising avenue for further investigation. It is difficult to control the initial bubble radius and strategies such as manipulating the dissolved gas concentration (by deaerating the process fluid) to prevent/control in-situ bubble nucleation and/or controlled sparging of bubbles may be explored to improve cavitation efficiency.

### 3.4 Other physical effects

**Fig. 10:**
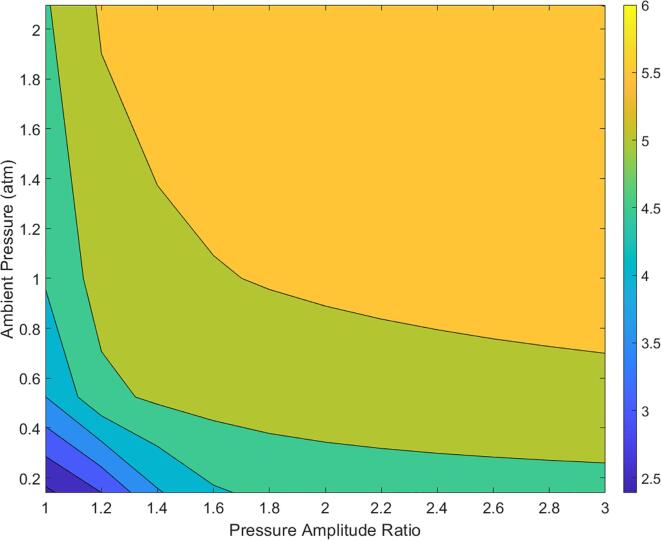
Contour plot of the logarithm of turbulent energy dissipation rate (m^2^/s^3^) as a function of the pressure amplitude ratio and the ambient pressure.

Besides the JHP and JV, it is possible to examine the physical effects of cavitation in different ways. For instance, the internal energy of the bubble at collapse conditions, which is later dissipated as shockwaves or a water jet may itself be used to assess the physical effects. In such a case, the internal energy at collapse may be obtained from single bubble dynamics simulations as shown in Fig. 7C. Another way to assess the physical effects is to track the turbulent stresses generated due viscous dissipation - eddies caused by an expanding and contracting bubble during cavitation [33]. The turbulent energy dissipation rate in this case may also be calculated by using the single bubble dynamics simulations as described by Sawant et. al. (2008) [33]. As discussed by Sawant et al., the length scale of the Eddie generated will be of the order of the radial fluctuation of the bubble. In the vicinity of bubble collapse, the liquid will be pulled by the shrinking bubble wall and hence the bubble wall velocity may be used to estimate the velocity scales. Based on these arguments, the average Eddie length was estimated by averaging the simulated radial trajectory (shown in Fig. 1) of the bubble. The fluctuating velocity in the liquid at a fixed distance of the maximum simulated bubble radius from the bubble center, was calculated using the radial velocities (available through the rate of change of the bubble radius solved in the present model) over the simulated lifetime of the bubble. These quantities were then used to obtain the turbulent energy dissipation rates (TEDR) (proportional to the velocity cubed divided by the characteristic Eddie length) [33], [61]. To illustrate such applications of the present model, the contour plot of the turbulent energy dissipation rate as a function of $P_{A}^{\text{'}}$ and the ambient pressure is shown in Fig. 10. The high turbulent energy dissipation rates realized by cavitation may explain why single passthrough cavitation device may result in significant reduction in droplet sizes of liquid – liquid emulsions [62].

### 3.5 Summary of results and potential applications

In the previous section two key quantities of interests – the •OH generation (relevant for quantifying potential of chemical effects) and the jet hammer pressure (relevant for quantifying potential of physical effects), were investigated. Influence of important operating parameters on these two quantities of interest were investigated over the ranges relevant to AC and HC. The key trends of the preceding analysis are summarised Table 4, Table 5. In general, the OHG may be promoted by increasing the ambient pressure, ambient temperature (within a certain range), decreasing the driving frequency and increasing the initial bubble radius for both HC and AC. Increasing the ambient temperature and the initial bubble radius have the added benefit of an earlier onset of cavitation for HC. The JHP may be promoted by increasing the ambient pressure, driving frequency and the initial bubble radius for both AC and HC. Increasing the initial bubble radius provides an additional benefit of an earlier onset of cavitation for HC. Thus, the model was useful in providing tangible guidelines for effecting process improvements for a wide range of cavitation processes and for identifying specific areas for further research.

**Table 4:** Summary of the major trends observed on increasing each of the operating parameters on the OHG and JHP for AC.

Operating Parameters	•OH generation (Chemical Effect)	Jet Hammer Pressure (Physical Effect)
$↑P_{\infty }$	Increases for all $P_{A}^{\text{'}}$	Has a minimum in terms of $P_{A}^{\text{'}}$; Increases at medium/high $P_{A}^{\text{'}}$
$↑T_{\infty }$	Increases for medium $P_{A}^{\text{'}}$	Increases at medium/high$P_{A}^{\text{'}}$
↑f	Decreases at medium/high $P_{A}^{\text{'}}$	Increases at low/medium $P_{A}^{\text{'}}$
$↑R_{0}$	Increases at lower $P_{A}^{\text{'}}$	Increases for all $P_{A}^{\text{'}}$

**Table 5:** Summary of the major trends observed on increasing each of the operating parameters on the OHG and JHP for HC.

Operating Parameters	•OH generation (Chemical Effect)	Jet Hammer Pressure (Physical Effect)
$↑P_{\infty }$	Increases for all $P_{A}^{\text{'}}$	Has a minimum in terms of $P_{A}^{\text{'}}$; Increases at medium/high $P_{A}^{\text{'}}$
$↑T_{\infty }$	Increases at low/intermediate$P_{A}^{\text{'}}$	Decreases at low $P_{A}^{\text{'}}$; enables earlier onset
↑f	Decreases for all $P_{A}^{\text{'}}$	Increases for al l$P_{A}^{\text{'}}$
$↑R_{0}$	Has an optimal value for all $P_{A}^{\text{'}}$	Increases for all $P_{A}^{\text{'}}$; enables earlier onset

As a word of caution, it should be noted that there are several complexities associated with cavitation processes besides those considered in the analysis of single bubble dynamics. For example, aspects such as the dependence on dissolved gas content, cloud formation [4], [63], attenuation of the pressure signal [50] etc. are known to complicate the cavitation process dynamics. However, despite such complications, the results and guidelines presented here would provide useful insights and will be useful in realizing further improvements in design and optimization of cavitation processes.

## 4 Conclusions

In the present work, key aspects of a single bubble cavitation dynamics model were investigated. The state-of-the-art single cavity dynamics models were reviewed, and suitable modifications were implemented to relax certain assumptions and to improve the mathematical rigour. The model was thoroughly verified by comparison against the previous state-of-the-art models. Important issues such as the onset of cavitation and chaotic behavior in single cavity dynamics were briefly addressed. The model was used to investigate the influence of operating conditions on the •OH generation (OHG) and the physical effects of cavitation like jet hammer pressure (JHP). The OHG was estimated by applying the minimization of Gibb’s free energy method using the model predicted pressure, temperature and composition at the collapse. A new equation was derived to estimate the JHP using the model predicted bubble collapse conditions. The effects of key operating parameters on the OHG, JHP and other characteristics used for quantifying physical effects of cavitation were investigated, and potential avenues for further research were identified.

The onset of cavitation was explained as a combination of the development of perturbations during the contraction phase at high pressure amplitude ratios and the bubble oscillations being in the unstable region as defined by the Blake threshold. The origin of chaotic behavior was shown to be a consequence of bubble ‘bouncing’ after the first contraction during one cycle interfering with the expansion during the next cycle. Across all simulations, it was found that the onset of cavitation was adequately captured using Blake threshold, with cavitation mathematically described as being a sharp and significant (orders of magnitude) increase in the bubble internal pressure and temperature. Simulations reaffirmed the previously reported collapse pressures of the order of 10,000 bar and temperatures of the order 5,000 K for varying operating conditions. The OHG and JHP due to cavitation were seen to be highly sensitive to ambient pressures and hence the effects of operating parameters were investigated separately for HC and AC.

Overall, an OHG value as high as 1x10^12^ •OH molecules per collapse event was observed under certain conditions in the present study. The OHG predicted by the present model agreed with those reported by Storey et. al. (2000) [7] though some mismatch was observed for the other chemical species involved. The reasons of the mismatch could not be confirmed. Both models present useful results and have their own merits in the face of significant uncertainties surrounding chemical kinetics at collapse conditions. The OHG was found to be significantly higher than the •H generation rate for all simulation cases, except for the lowest driving frequency (5 kHz) in HC, wherein the collapse temperature was upwards of 10,000 K. For the case of HC, an optimum bubble size of 20 μ*m* was found for maximizing the OHG.

The predicted JHP were within a range of ~ 100 to 1000 bar under varying operating conditions. The JHP was found to go through a minimum with respect to the pressure amplitude ratio in most cases considered. Specific trends with regards to key operating parameters were summarized in Table 4, Table 5. Specific guidelines provided in the present study would be useful for the optimization of cavitation processes spanning a broad range of applications. The results obtained from the presented model will also be useful as a closure model to represent micro-scale cavity collapse phenomena in macro-scale reaction engineering and/ or computational fluid dynamics models describing cavitation devices and cavitation processes.

## CRediT authorship contribution statement

**Ajinkya V. Pandit:** Investigation, Methodology, Formal analysis, Validation, Writing – original draft. **Varaha P. Sarvothaman:** Investigation, Methodology, Validation. **Vivek V. Ranade:** Conceptualization, Methodology, Funding acquisition, Supervision, Writing – review & editing.

## Declaration of Competing Interest

The authors declare that they have no known competing financial interests or personal relationships that could have appeared to influence the work reported in this paper.
