# Supplementary material for: Estimation of chemical and physical effects of cavitation by analysis of cavitating single bubble dynamics
Source: Ultrason Sonochem. 2021 Jul 23;77:105677. doi: 10.1016/j.ultsonch.2021.105677 (PMC8339230; doi:10.1016/j.ultsonch.2021.105677)
Supplement: Supplementary data 1 [file mmc1.pdf]

Estimation of Chemical and Physical Effects of Cavitation by Analysis of Cavitating Single Bubble Dynamics

Ajinkya V. Pandit[a], Varaha P. Sarvothaman[a] and Vivek V. Ranade*[a,b]

[a] School of Chemistry and Chemical Engineering, Queen's University, Belfast, UK

[b] Bernal Institute, University of Limerick, Limerick, Ireland

* Email Address: v.ranade@qub.ac.uk, vivek.ranade@ul.ie

Supplementary Information

A1. Equation for dP^B/dt

The derivation for the equation of rate of change of bubble internal pressure assuming a VdW EoS is given below. Either of Equations A1.3 or 1.4 may be used.

$$P^B = \frac{N_t^B k\, T^B}{\frac{4\pi}{3}R^3 - \frac{N_t^B}{N_A} b} - a \frac{\left(\frac{N_t^B}{N_A}\right)^2}{\left(\frac{4\pi}{3}R^3\right)^2} \qquad \text{A1.1}$$

$$a = \sum_i a_i {x_i^B}^2 \,;\; b = \sum_i x_i^B b_i$$

$$\frac{dP^B}{dt} = \frac{\left(\frac{4\pi}{3}R^3 - \frac{N_t^B}{N_A}\sum_i x_i^B b_i\right)\frac{d}{dt}\left(N_t^B k\, T^B\right) - \left(N_t^B k\, T^B\right)\frac{d}{dt}\left(\frac{4\pi}{3}R^3 - \frac{N_t^B}{N_A}\sum_i x_i^B b_i\right)}{\left(\frac{4\pi}{3}R^3 - \frac{N_t^B}{N_A}\sum_i x_i^B b_i\right)^2} - \sum_i 2\, a_i \frac{\left(\frac{N_i^B}{N_A}\right)}{\left(\frac{4\pi}{3}R^3\right)}\left[\frac{\frac{4\pi}{3}R^3 \frac{d}{dt}\left(\frac{N_i^B}{N_A}\right) - \left(\frac{N_i^B}{N_A}\right)4\pi R^2 \frac{dR}{dt}}{\left(\frac{4\pi}{3}R^3\right)^2}\right] \qquad \text{A1.2}$$

$$\frac{dP^B}{dt} = \frac{\left(\frac{4\pi}{3}R^3 - \frac{N_t^B}{N_A}\sum_i x_i^B b_i\right)\left(k\, T^B \frac{dN_t^B}{dt} + N_t^B k \frac{dT^B}{dt}\right) - \left(N_t^B k\, T^B\right)\left(4\pi R^2 \frac{dR}{dt} - \sum_i \frac{b_i}{N_A}\frac{d x_i^B N_t^B}{dt}\right)}{\left(\frac{4\pi}{3}R^3 - \frac{N_t^B}{N_A} b\right)^2} + 6a \frac{\left(\frac{N_t^B}{N_A}\right)^2}{\left(\frac{4\pi}{3}R^3\right)^2}\frac{1}{R}\frac{dR}{dt} - \sum_i \left(\frac{2a_i}{N_A}\right)\frac{\left(\frac{N_i^B}{N_A}\right)}{\left(\frac{4\pi}{3}R^3\right)^2}\frac{dN_i^B}{dt} \qquad \text{A1.3}$$

$$\frac{dP^B}{dt} = \frac{\left(\frac{4\pi}{3}R^3 - \frac{N_t^B}{N_A}\sum_i x_i^B b_i\right)\left(k\, T^B \frac{dN_t^B}{dt} + N_t^B k \frac{dT^B}{dt}\right) - \left(N_t^B k\, T^B\right)\left(4\pi R^2 \frac{dR}{dt} - \sum_i \frac{b_i}{N_A}\frac{dN_i^B}{dt}\right)}{\left(\frac{4\pi}{3}R^3 - \frac{N_t^B}{N_A} b\right)^2} + \sum_i 6a_i \frac{\left(\frac{N_i^B}{N_A}\right)^2}{\left(\frac{4\pi}{3}R^3\right)^2}\left(\frac{1}{R}\frac{dR}{dt}\right) - \sum_i \left(\frac{2a_i}{N_A}\right)\frac{\left(\frac{N_i^B}{N_A}\right)}{\left(\frac{4\pi}{3}R^3\right)^2}\frac{dN_i^B}{dt} \qquad \text{A1.4}$$

A2. Auxiliary Equations for the closure of the Mass Transfer Model

The following Equations A2.1 – A2.15 are required for the closure of the mass transfer model described in Section 2.3 and were derived using the molecular theory of thermodynamics.

$$C_i^B = \frac{N_i^B}{V^B}; N_t^B = \sum_{i=1}^{N_c} N_i^B \,; x_i^B = \frac{N_i^B}{N_t^B} \tag{A2.1}$$

$$P_{v,w\ or\ volatile}^i = (x_{volatile}^{\infty})\, P_{v,volatile}^{\infty} \tag{A2.2}$$

$$C_{w\ or\ volatile}^i = \frac{P_{v,w\ or\ volatile}^i}{k\, T^{\infty}} \tag{A2.3}$$

$$P_{v,volatile}^t = \sum_{i=1}^{N_c} P_{v,w\ or\ volatile}^i \tag{A2.4}$$

$$P_{i \neq volatile}^i = \left(P^B - P_{v,volatile}^t\right) x_i^B \tag{A2.5}$$

$$C_{i \neq volatile}^i = \frac{P_{i \neq volatile}^i}{k\, T^{\infty}} \tag{A2.6}$$

$$C_t^i = \sum_{i=1}^{N_c} C_i^i \,;\; x_i^i = \frac{C_i^i}{C_t^i} \tag{A2.7}$$

$$\frac{1}{D_i} = \sum_{j \neq i} \frac{x_j^B}{(1 - x_i^B)\, D_{i,j}} \tag{A2.8}$$

$$D_{i,j} = \frac{3}{8} \frac{\sqrt{\frac{\pi\, k\, T^{\infty}}{\Gamma_{i,j}}}}{C_{i,j}^i\, \pi\, \alpha_{i,j}^2\, \Omega_{i,j}^{(1,1)}} \tag{A2.9}$$

$$\Gamma_{i,j} = \frac{2\, m_i\, m_j}{m_i + m_j} \tag{A2.10}$$

$$m_i = \frac{Mw_i}{N_{av}} \tag{A2.11}$$

$$C_{i,j}^i = C_i^i + C_j^i \tag{A2.12}$$

$$\alpha_{i,j} = \frac{\alpha_i + \alpha_j}{2} \tag{A2.13}$$

$$\Omega_{i,j}^{(1,1)} = \left[-34.8998 + \frac{0.69463}{T_{i,j}^{*0.825232}} + \frac{35.63592}{T_{i,j}^{*0.00146}}\right] \tag{A2.14}$$

$$T_{i,j}^* = \frac{T^{\infty}}{\sqrt{\psi_i \psi_j}} \tag{A2.15}$$

A3. Equation for $C_{v,mix}^{*}$

The generalized energy balance equation may be formally written as Equation A3.1:

$$\frac{d}{dt}\left(\sum_{i=1}^{N_c} C_{v,i}\, N_i^B\, T^B\right) = \left[\left(-4\,\pi\, R^2\, \lambda\, \left(\frac{\partial T}{\partial r}\right)_{r=R}\right) - \left(4\,\pi\, R^2 P^B\, \frac{dR}{dt}\right) + \left(\sum_{i=1}^{N_c} (h_i)\, \frac{dN_i^B}{dt}\right)\right] \quad \text{A3.1}$$

The left-hand side of the equation may be expanded as Equation A3.2:

$$\frac{d}{dt}\left(\sum_{i=1}^{N_c} C_{v,i} N_i^B T^B\right) = \frac{dT^B}{dt}\left(\sum_{i=1}^{N_c} C_{v,i} N_i^B\right) + T^B\left(\sum_{i=1}^{N_c} N_i^B \frac{dC_{v,i}}{dt}\right) + T^B\left(\sum_{i=1}^{N_c} C_{v,i} \frac{dN_i^B}{dt}\right) \quad \text{A3.2}$$

The derivation for the rate of change of the $C_{v,i}$ is given as follows in Equations A3.3 – A3.8:

$$\frac{d}{dt}\left(\sum_{i=1}^{N_c} C_{v,i} N_i^B T^B\right) = \frac{dT^B}{dt}\left(\sum_{i=1}^{N_c} C_{v,i} N_i^B\right) + T^B\left(\sum_{i=1}^{N_c} N_i^B \frac{dC_{v,i}}{dt}\right) + T^B\left(\sum_{i=1}^{N_c} C_{v,i} \frac{dN_i^B}{dt}\right) \quad \text{A3.3}$$

$$= k\sum_{j=1}^{N_\theta} \frac{\left(exp(\delta_{i,j}) - 1\right)^2 \frac{d\left(\delta_{i,j}^2\, exp(\delta_{i,j})\right)}{dt} - \delta_{i,j}^2\, exp(\delta_{i,j}) \frac{d\left(exp(\delta_{i,j}) - 1\right)^2}{dt}}{\left(exp(\delta_{i,j}) - 1\right)^4} \quad \text{A3.4}$$

$$= k\sum_{j=1}^{N_\theta} \frac{\left(exp(\delta_{i,j}) - 1\right)^2 \left[\delta_{i,j}^2 \frac{d\left(exp(\delta_{i,j})\right)}{dt} + exp(\delta_{i,j}) \frac{d(\delta_{i,j}^2)}{dt}\right] - \delta_{i,j}^2\, exp(\delta_{i,j}) \left[2\left(exp(\delta_{i,j}) - 1\right) \frac{d\left(exp(\delta_{i,j}) - 1\right)}{dt}\right]}{\left(exp(\delta_{i,j}) - 1\right)^4} \quad \text{A3.5}$$

$$= k\sum_{j=1}^{N_\theta} \frac{\left(exp(\delta_{i,j}) - 1\right)^2 \left[\delta_{i,j}^2 exp(\delta_{i,j}) \frac{d(\delta_{i,j})}{dt} + 2\delta_{i,j} exp(\delta_{i,j}) \frac{d(\delta_{i,j})}{dt}\right] - \delta_{i,j}^2\, exp(\delta_{i,j}) \left[2\left(exp(\delta_{i,j}) - 1\right) exp(\delta_{i,j}) \frac{d(\delta_{i,j})}{dt}\right]}{\left(exp(\delta_{i,j}) - 1\right)^4} \quad \text{A3.6}$$

$$= k\sum_{j=1}^{N_\theta} \frac{d(\delta_{i,j})}{dt} \frac{\left\{\left(exp(\delta_{i,j}) - 1\right)^2 \left[\delta_{i,j}^2 exp(\delta_{i,j}) + 2\delta_{i,j} exp(\delta_{i,j})\right] - \delta_{i,j}^2\, exp(\delta_{i,j}) \left[2\left(exp(\delta_{i,j}) - 1\right) exp(\delta_{i,j})\right]\right\}}{\left(exp(\delta_{i,j}) - 1\right)^4} \quad \text{A3.7}$$

$$= k\sum_{j=1}^{N_\theta} -\frac{\theta_{i,j}}{T^{B^2}} \frac{dT^B}{dt} \frac{\left\{\left(exp(\delta_{i,j}) - 1\right)^2 \left[\delta_{i,j}^2 exp(\delta_{i,j}) + 2\delta_{i,j} exp(\delta_{i,j})\right] - \delta_{i,j}^2\, exp(\delta_{i,j}) \left[2\left(exp(\delta_{i,j}) - 1\right) exp(\delta_{i,j})\right]\right\}}{\left(exp(\delta_{i,j}) - 1\right)^4} \quad \text{A3.8}$$

After some simplifications, the following equation (Equation A3.9) may be obtained for the rate of change of $C_{v,i}$:

$$\therefore \frac{dC_{v,i}}{dt} = -\frac{1}{T^B} \frac{dT^B}{dt} \Psi_i;\; \Psi_i = k\sum_{j=1}^{N_\theta} \delta_{i,j} \frac{\left\{\left(exp(\delta_{i,j})\right)^2 (2\delta_{i,j} - \delta_{i,j}^2) - (\delta_{i,j}^2 + 2\delta_{i,j}) exp(\delta_{i,j})\right\}}{\left(exp(\delta_{i,j}) - 1\right)^3} \quad \text{A3.9}$$

$$\therefore \frac{d}{dt}\left(\sum_{i=1}^{N_c} C_{v,i} N_i^B T^B\right) = \frac{dT^B}{dt}\left(\sum_{i=1}^{N_c} (C_{v,i} - \Psi_i) N_i^B\right) + T^B\left(\sum_{i=1}^{N_c} C_{v,i} \frac{dN_i^B}{dt}\right) \quad \text{A3.10}$$

Substituting this Equation into the generalized energy balance equation and after rearranging, we may obtain the equation for the rate of change in bubble temperature as Equation A3.11:

$$\frac{dT^B}{dt} = \frac{1}{\sum_{i=1}^{N_c} (C_{v,i} - \Psi_i) N_i^B} \left[\left(-4\,\pi\, R^2\, \lambda\, \left(\frac{\partial T}{\partial r}\right)_{r=R}\right) - \left(4\,\pi\, R^2 P^B\, \frac{dR}{dt}\right) + \left(\sum_{i=1}^{N_c} \left(h_i - T_B C_{v,i}\right) \frac{dN_i^B}{dt}\right)\right] \quad \text{A3.11}$$

The term Ψ_i used in the above equation was implicitly neglected as reported by Toegel et. al. [20] and both the terms Ψ_i and $T_B C_{v,i}$ were neglected by Chakma et. al. [22].

A4. Auxiliary Equations for the closure of the Heat Transfer Model

The following Equations A4.1 – A4.11 are required for the closure of the heat transfer model described in Section 2.4 and were derived using the molecular theory of thermodynamics.

$$\kappa = \frac{\lambda_{mix}}{\rho_{mix} C_{p,mix}} \tag{A4.1}$$

$$\lambda_{mix} = \sum_{i} \frac{x_i^B \lambda_i}{\sum_j x_i^B \beta_{i,j}} \tag{A4.2}$$

$$\lambda_i = \frac{15k}{m_i} \eta_i \left(\frac{4f_i}{30} + \frac{3}{5} \right) \tag{A4.3}$$

$$\eta_i = \frac{5}{16} \frac{\sqrt{\pi \, m_i \, k \, T^{\infty}}}{\pi \, \alpha_i^2 \, \Omega_{i,i}^{(2,2)}} \tag{A4.4}$$

$$\Omega_{i,i}^{(2,2)} = \left[-34.90317 + \frac{0.83841}{T_i^{*0.73653}} + \frac{35.6341}{T_i^{*0.00109}} \right] \tag{A4.5}$$

$$T_i^* = \frac{T^{\infty}}{\psi_i} \tag{A4.6}$$

$$\beta_{i,j} = \frac{1}{\sqrt{8 \left(1 + \frac{m_i}{m_j} \right)}} \left[1 + \left(\frac{\eta_i}{\eta_j} \right)^{-0.5} \left(\frac{m_i}{m_j} \right)^{0.25} \right]^2 \tag{A4.7}$$

$$C_{v,i} = k \left(\frac{f_i}{2} + \sum_{j=1}^{N_\theta} \frac{\delta_{i,j}^2 \, exp(\delta_{i,j})}{\left(exp(\delta_{i,j}) - 1 \right)^2} \right); \; \delta_{i,j} = \frac{\theta_{i,j}}{T^B} \tag{A4.8}$$

$$C_{p,i} = k \left(\frac{f_i + 2}{2} \right) \tag{A4.9}$$

$$\rho_{mix}^B \, C_{p,mix} = \sum_{i=1}^{N_c} C_{p,i} \;\; C_i^B \tag{A4.10}$$

$$h_i = C_{p,i} \, T^{\infty} \tag{A4.11}$$

A5. Verification of Model

To verify the model code, simulations were performed for a base case using both the Van der Waal's (VdW) and the Ideal Gas (IG) equation of states (EoSs) in the present model. A comparison between the bubble radius profile and the number of water molecules profile using the four models (VdW, IG, Storey et. al. (2000) and Toegel et. al. (2000)) is shown in Figure A5.1. The results indicate excellent agreement with previous models and show that there is no effect of the EoS assumption on the simulation results for the bubble radius and number of water molecules' profiles.

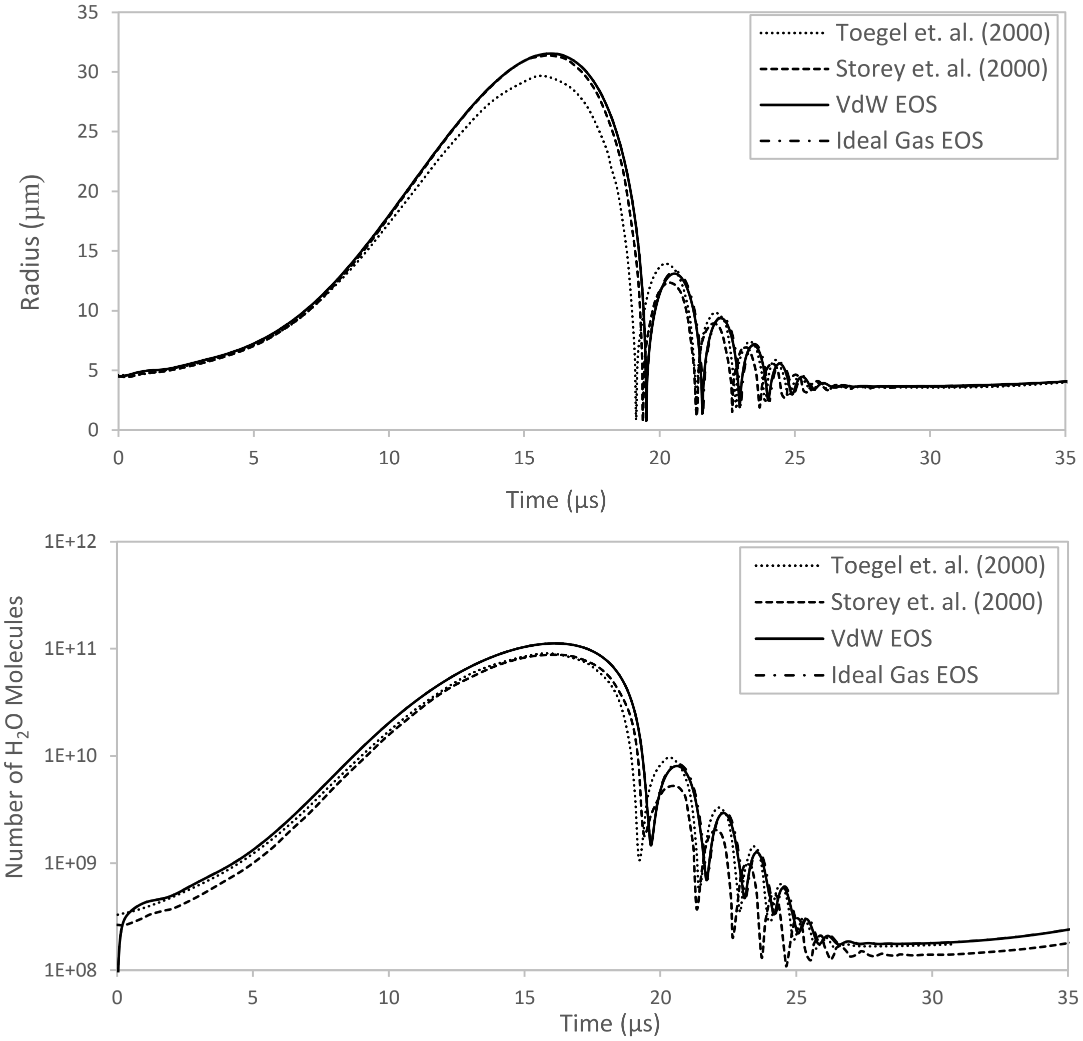

Figure A5.1: Comparison between model predictions using Toegel et. al. (2000), Storey et. al. (2000), VdW EOS and Ideal Gas models for temporal profiles of (a) bubble radius and (b) number of water molecules

The data regarding temperature was not available for Toegel et. al. [20]. Storey et. al. [7] considered the radial variation in temperature and reported significant radial variation, especially around the instance of collapse. Conversely in the present model, the contents of the bubble were assumed to be perfectly mixed. Hence, it is expected that the perfectly mixed temperature, which is averaged over all the bubble contents, would be significantly lower than the temperature at the central point as

reported by Storey et. al. [7]. Even then, such a comparison as shown in Figure A5.2 enabled the verification of the key time scales involved and confirms the order of magnitude of the temperature rise. The time of collapse was defined as the first time that the bubble reached the minimum value after a contracting rapidly from the maximum size at a time of around 19µs as seen in Figure A5.1. The pressure and temperature were seen to be only marginally affected by the choice of the EoS as seen in Figure A5.2.

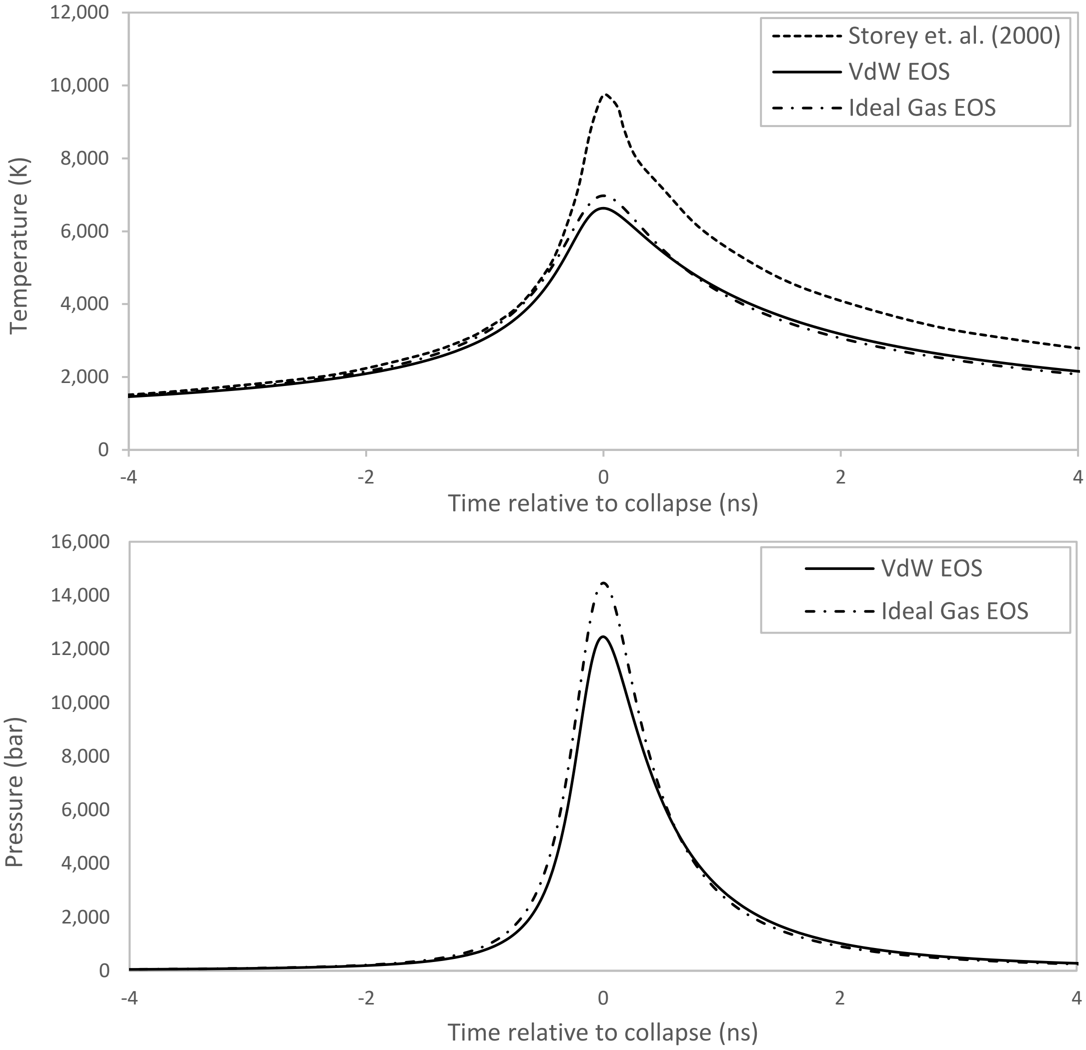

Figure A5.2: Comparison between model predictions for temporal profiles around the time of collapse of (a) bubble internal temperature using Storey et. al. [7], VdW EOS and Ideal Gas models and (b) bubble internal pressure using VdW EOS and Ideal Gas models

A comparison was also performed for the water content at collapse for various conditions and simulation ns revealed a good agreement with previously reported values as shown in Table A5.1. As seen previously, there was no effect of the choice of EoS on the water content at collapse. A comparison could not be made with regards to the pressure profiles as they were not reported by both Storey et. al. [7] or Toegel et. al. [20].

Table A5.1: Comparison between predictions using different models for the percentage number of water molecules in the bubble at collapse

Case	R0 (μm)	Pa (bar)	T (K)	f (Hz)	Moss et. al. [36]	Storey et. al. [7]	Toegel et. al. [20]	VdW EOS	Ideal Gas EOS
A1	6	1.4	293.15	20.6	40	33	33	41	41
B1	4	1.32	293.15	20.6	36	27	23	31	31
C1	2.1	1.29	293.15	20.6	30	22	14	21	21

A6. Analysis of Contributing Terms for Cavitation Inception

For a deeper understanding about how the inception of cavitation is occurring, the contributing terms of the model equations were looked at in more detail as a function of the pressure amplitude ratio (P_A'). The dimensional equations for the bubble dynamics used in the present study as given by Equation (A6.1) and (A6.2).

$$\frac{dR}{dt} = s \qquad \text{A6.1}$$

$$\frac{ds}{dt} = \underset{(T1)}{\left(\frac{\emptyset_3\,(P^B - P^\infty)}{\emptyset_1 \rho R}\right)} + \underset{(T2)}{\left(\frac{1}{\emptyset_1 \rho c}\right)\frac{dP^B}{dt}} - \underset{(T3)}{\left(\frac{4\,\nu\,s}{\emptyset_1\,R^2}\right)} - \underset{(T4)}{\left(\frac{2\,\sigma}{\emptyset_1\,\rho\,R^2}\right)} - \underset{(T5)}{\left(\frac{1.5\,\emptyset_2\,\,s^2}{\emptyset_1\,R}\right)} \qquad \text{A6.2}$$

As can be seen from the above equation, the rate change of the bubble radius velocity is represented by 5 terms which will be referred to hereafter as T1, T2 and so on till T5 as given by Equation (A6.2). For P_A' values below the cavitation threshold, it was observed that T1 and T4 are the major terms governing the dynamics of the cavitation as shown in Figure A6.1 (a) ($P_A' = 0.8$). The rest of the terms were negligible and may be ignored. It should be noted that the Keller Miksis correction terms ($\emptyset_i$) for a region around the cavitation threshold are close to unity and as the present analysis deals with the inception of cavitation, they may be ignored. The initial perturbations to T1 shown in Figure A6.1 (a) correspond to the sharp initial influx of water molecules and do not impact the bubble dynamics above the cavitation threshold. T1 may be considered as the pressure difference term and T4 the surface tension term and hence the bubble dynamics for sub cavitation regimes is governed by the pressure difference and surface tension.

As the P_A' was increased to a value of 1.0, cavitation was observed as was described in Section 3.1 of the manuscript. It was seen from Figure A6.1 (b) ($P_A' = 1$) that for this period, the term T5 was the first significant negative contributing term which offset the system and led to the bubble contraction. The term T1 only increased to compensate for the decrease in T5 around the time of collapse. The rest of the terms i.e. T2 (rate of change of internal bubble), T3 (viscous dissipation) and T4 (surface tension) were not significant from the point of view of cavitation. Hence the term T5, which can be considered as sort of a kinetic energy, was deemed responsible for the onset of the cavitation. It is interesting to note that higher the absolute value of contribution for T5, in the absence of a counter balancing force, the more accelerated is the bubble contraction which in turn increases the absolute value contribution of T5. This compounding effect of increasing T5 which leads to an increasingly accelerated contraction is only offset when the pressure inside the bubble increases significantly to counteract the contraction of the bubble.

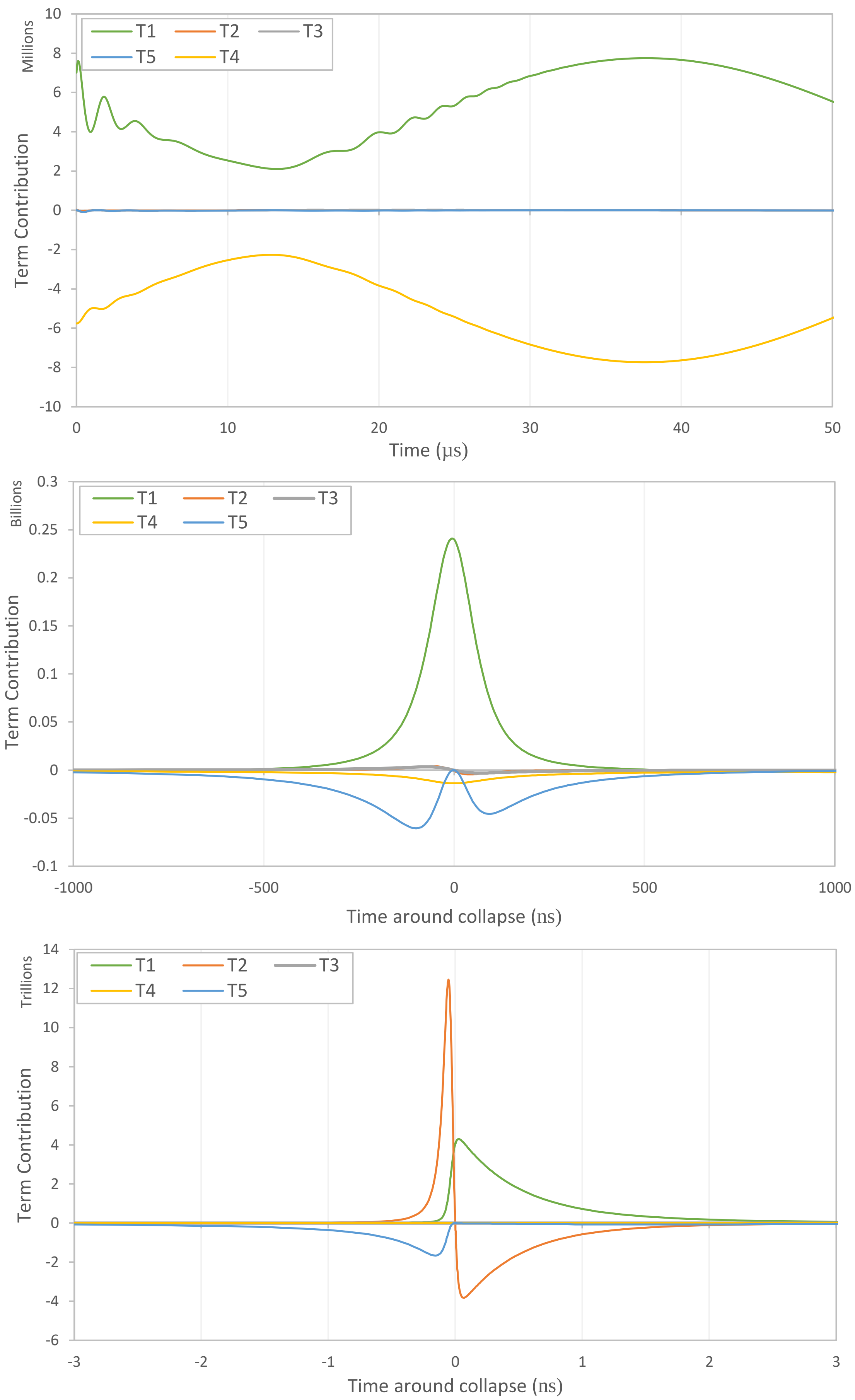

Figure A6.1: Contributing terms for the Keller-Miksis equation (a) $P_A' = 0.8$ (b) $P_A' = 1.0$ (c) $P_A' = 1.2$

On further increasing P_A' as shown in Figure A6.1 (c) ($P_A' = 1.2$), T5 was still the term which caused the offset leading to the accelerated contraction of the bubble. The absolute value of T5 was seen to be several orders of magnitude higher than that for when P_A' was equal to 1.0. It was observed that for this case, T2 (rate of change of bubble internal pressure) was the term which first increased to counteract the contraction due to T5. However, this counteraction did not occur until with 1ns before the point of minimum contraction as opposed to several hundred ns as for the case of $P_A' = 1.0$. The pressure then increased significantly enough to counteract the bubble contraction which was indicated by an increase in T1 and a decrease in T2. The period after the collapse is also shown for all cases, however, is not of immediate interest was shown only for the sake of completeness. An interesting feature of the present analysis is that the counteraction term changes from T1 when $P_A' = 1.0$ to T2 when $P_A' = 1.2$. This suggests an inflection point in a bubble dynamics for a P_A' value between 1.0 to 1.2 which was clearly seen in the collapse temperature graph shown in Figure A7.1 in Section A7 in the Supporting Information. Presently, the physical significance of this transition is not clear.

A7. Chaos in Cavitation

The pressure amplitude ratio (P_A^*) was chosen as the key independent variable spanning a range over which the sensitivity of the simulation results was investigated. Simulations for the sensitivity analysis were performed spanning five oscillation cycles of the driving sinusoidal pressure wave unless otherwise specified. The maximum pressure and temperature for each of the sensitivity analysis simulations was plotted versus P_A^* as shown in Figure A7.1. It was observed that after a certain P_A^*, simulation results started to deviate from a well-behaved nature displayed by simulation results for a single cycle and showed an erratic pattern as seen from Figure A7.1. Such a deviation was akin to chaotic behaviour previously reported for cavitation. For further analysis of this erratic behaviour, single bubble simulations were performed for a value of P_A^* after which the chaotic behaviour was observed ($P_A^* \sim 2.25$). For simulations spanning a single driving pressure cycle as shown in Figure A7.2 (a), it was observed that regardless of the pressure, there was a bubble oscillation period after the first contraction which was seen to be akin to a bouncing ball before an equilibrium or initial state was achieved.

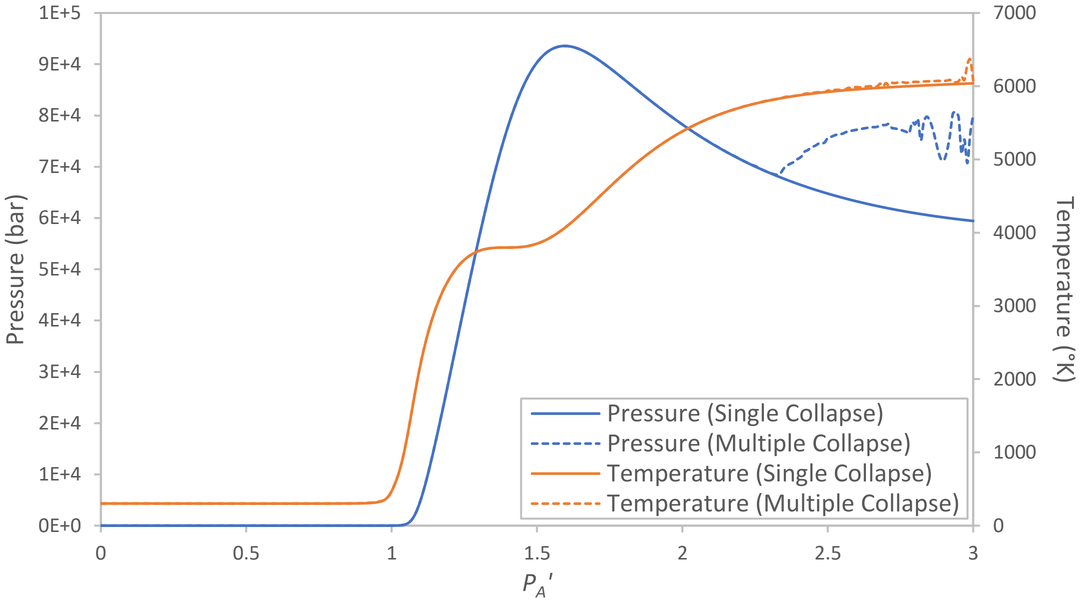

Figure A7.1: Collapse pressure and temperature versus P_A' assuming single collapse and multiple collapse condition

On increasing P_A^*, the period to reach the initial state was seen to extend beyond the duration of the cycle which was primarily due to the extended expansion of the bubble. On performing the single bubble simulations for multiple cycles with P_A^* = 3, it was observed that the non-equilibrium or bouncing state of the first cycle of the bubble interfered with the expansion during the second cycle. the second cycle. This complex interaction which could be discerned from the difference between the bubble trajectories for a single cycle versus multiple cycles as shown in Figure A7.2 (a), led to a highly non-linear and chaotic behaviour. It was observed that on performing the simulations for multiple

cycles, a new steady state was reached with the bubble radius reaching a maximum radius value as much as twice the initial and required close to fifteen cycles to set in as shown in Figure A7.2 (b). The maximum temperatures and pressures were also thus changed and varied depending duration of the simulation until the steady state was reached as a result of the chaos resulting from the driving pressure frequency and the bubble bouncing frequency.

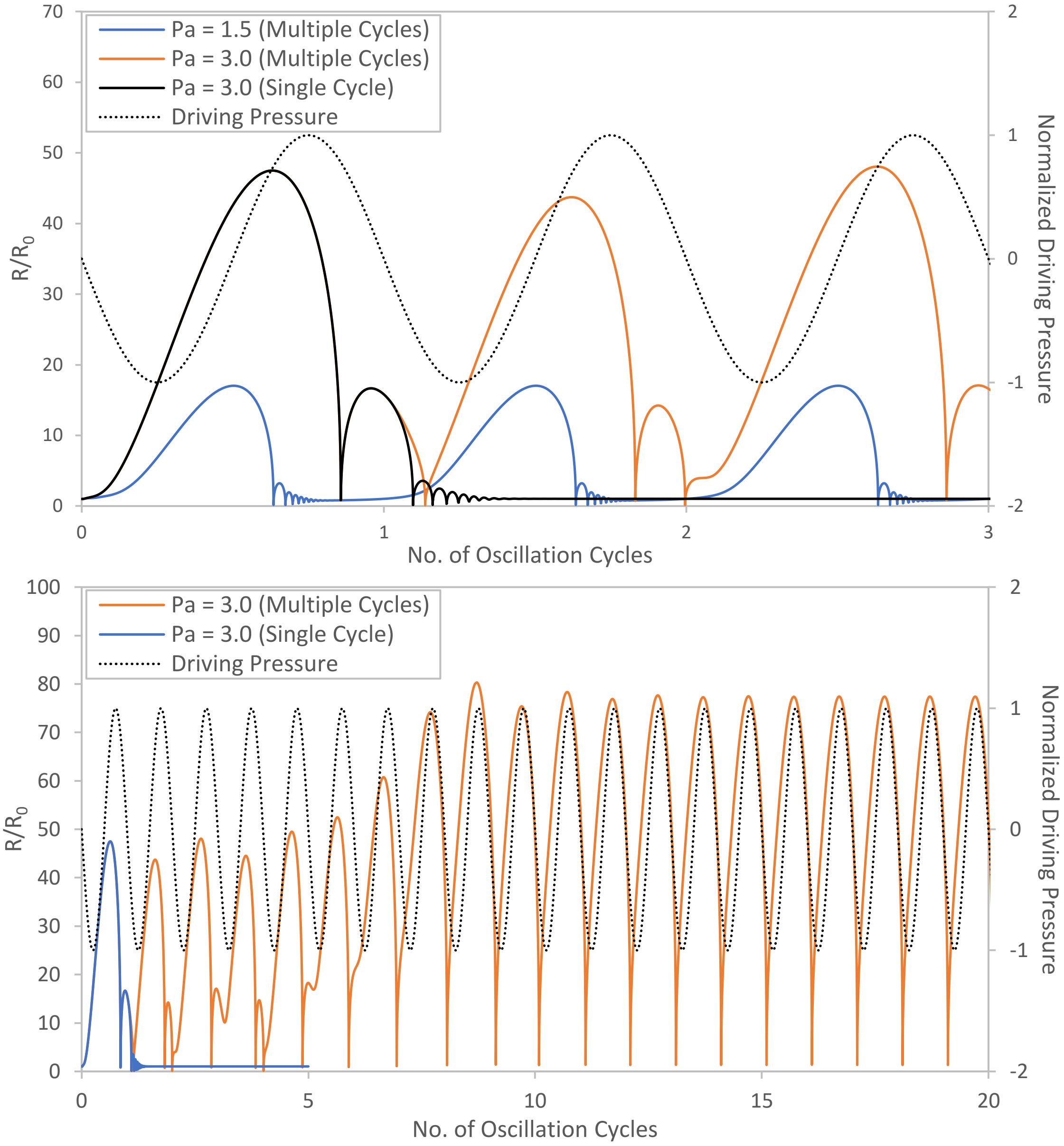

Figure A7.2: (a) Comparison between bubble radius profiles for $P_A' = 1.5$ & 3 pressure amplitude ratios assuming single collapse and multiple collapse conditions

A8. Sensitivity Analysis of Collapse Pressure and Temperature

The sensitivity analysis results of the collapse pressures and temperatures for different operating parameters are presented in this section. The initial abrupt changes which are observed the collapse pressure profiles (for example in Figure A8.1) at a pressure amplitude ratio lesser than the Blake's threshold pressure are numerical in nature. The automatic solver detection of the collapse event was defined as when the bubble radial velocity becomes zero and the direction of the rate of change of the velocity was positive. For low P_A' values, the 'collapse' event was erroneously detected as being the perturbation caused by the initial influx of water resulting in a pressure close to the initial pressure as visible in the Figure A8.1 for the case of $P_\infty = 15P_v$. On increasing P_A' (below the Blake's threshold), the 'collapse' instance is detected as the instance when the bubble reaches a minimum size during a contraction-expansion cycle which mirrors the driving pressure signal as shown in Figure A8.1 for $P_A' = 0.8$. The pressure in this case increases and hence a sharp jump is observed in the pressure values corresponding to a switching between the detection of the initial perturbation of water to the point of minimum radius. On further increasing P_A' to values near (still below) the Blake's threshold, perturbations as described in Section 3.1 in the manuscript are observed during a contraction phase of the bubble as shown in Figure 1 (Section 3.1) for $P_A' = 0.9$. The 'collapse' instance is thus numerically detected as the instance when the perturbations first start to become significant. As the bubble is near the maximum size, the pressure is reduced and an abrupt reduction in the collapse pressure values is observed. On further increasing P_A', as the perturbations develop into cavitation as described in Section 3.1 and the collapse event is detected accurately using the numerical criteria specified previously. These changes are not visible on a linear scale, however, were retained as they point towards key changes in the bubble dynamics, especially with regards to the onset of the perturbations.

Another noteworthy detail which is visible across all the sensitivity analysis studies is an intermediate plateau observed in the collapse temperature profile after the Blake's threshold pressure. It was speculated in Section A6 in the Annexure that the intermediate plateau occurs due to a switching of prominence between two terms, as the dominant term which counterbalances the contraction term in the model equations. The physical significance of this transition is presently not certain.

A8.1. Ambient Pressure

As described in Section 3.1, the ambient pressure conditions may vary significantly depending upon the mode of cavitation chosen. Further, the ambient pressure may also be considered as an operating parameter which may significantly influence the process dynamics. The contour plots for the collapse pressure and temperature on the ambient pressure were shown in Figure A8.1 wherein collapse

pressure values of the order of 10^4 to 10^5 bar and collapse temperature values in the order of 5,000 K were observed.

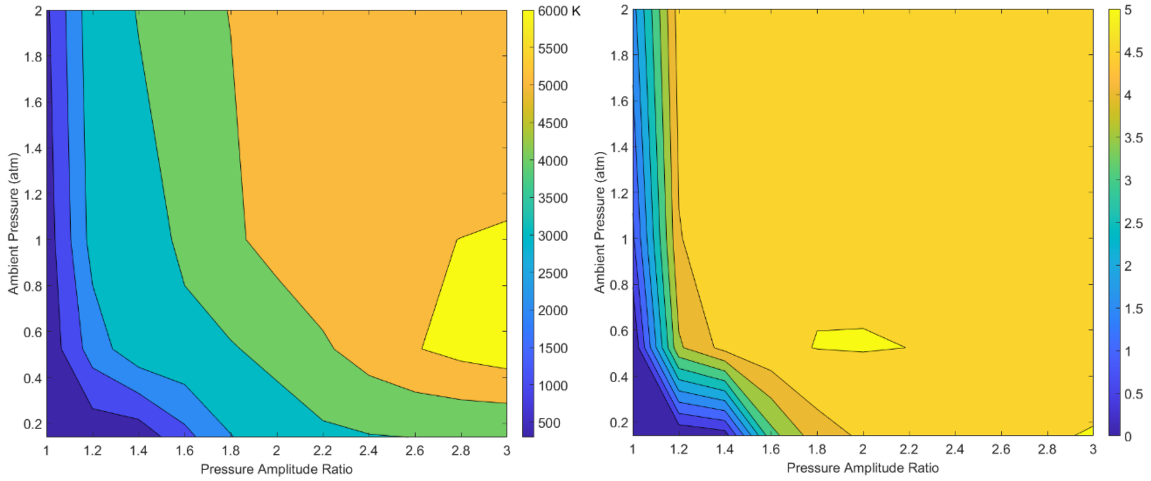

Figure A8.1: Comparison between the (a) collapse temperature (b) logarithm of collapse pressure versus P_A' profiles for varying values of ambient pressure

A8.2. Ambient Temperature for Acoustic Cavitation

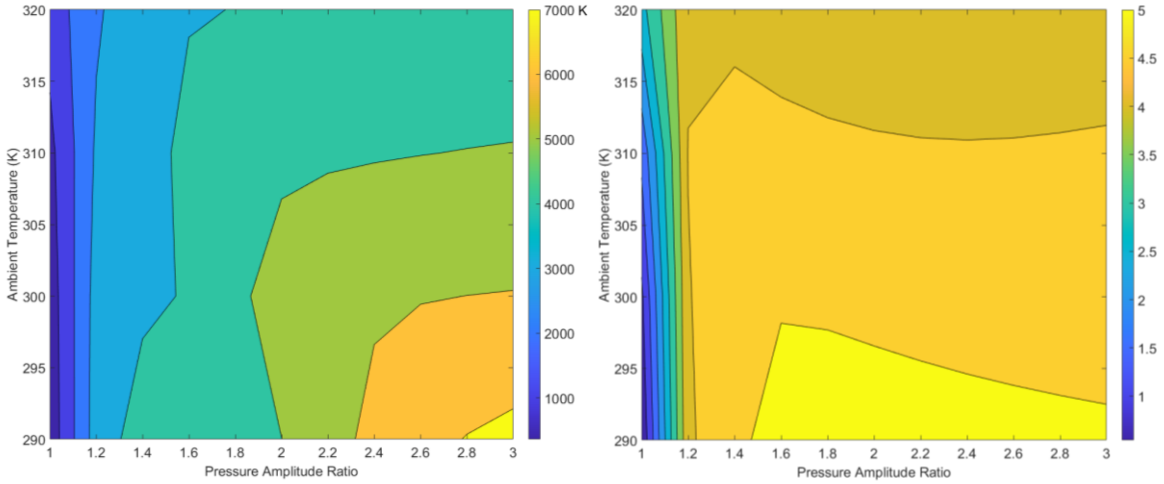

Figure A8.2: Comparison between the (a) collapse temperature (b) logarithm of collapse pressure versus P_A' profiles for varying values of ambient temperature for acoustic cavitation

The ambient temperature may also be used as an important operating parameter most importantly due to the dependence of reaction rates and vapor pressure on temperature. The contour plots for the collapse pressure and temperature on the ambient temperature were shown in Figure A8.2 wherein collapse pressure values of the order of 10^4 to 10^5 bar and collapse temperature values in the order of 5,000 K were observed. The collapse pressures were seen to decrease (by an order of ~10^4 bar) and the collapse temperatures were also seen to decrease (by an order of 1,000 K) with increasing ambient temperatures at higher values of P_A'.

A8.3. Ambient Temperature for Hydrodynamic Cavitation

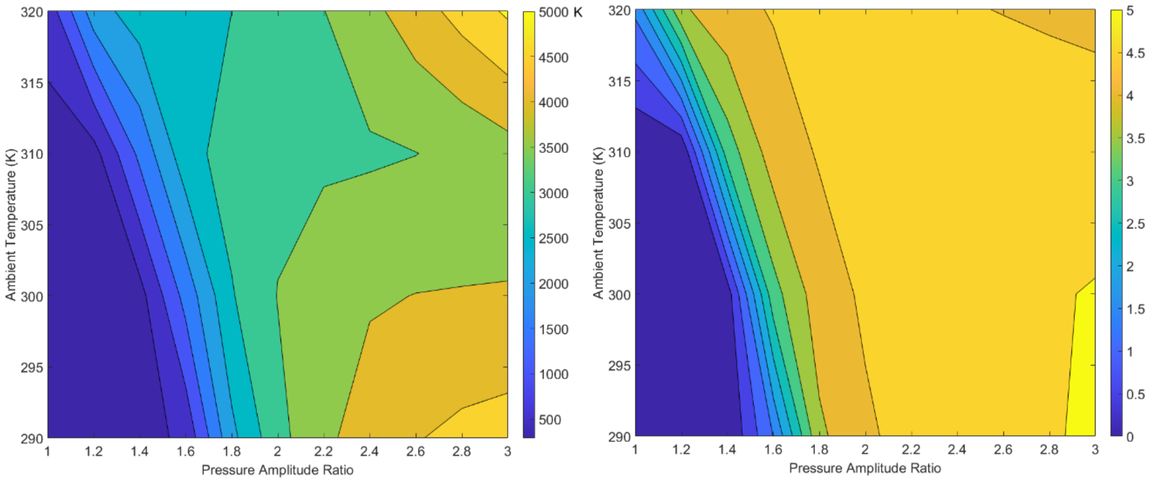

Figure A8.3: Comparison between the (a) collapse temperature (b) logarithm of collapse pressure versus P_A' profiles for varying values of ambient temperature for hydrodynamic cavitation

The onset of cavitation occurred earlier with increasing temperatures as seen in Figure A8.3. Collapse pressure values of the order of 10^4 to 10^5 bar and collapse temperature values in the order of 5,000 K were observed. A non-linear trend was seen for the collapse pressures and temperatures at higher values of P_A'.

A8.4. Driving Frequency for Acoustic Cavitation

The effects of the driving frequency on the peak pressures and temperatures for acoustic cavitation are shown in Figure A8.4. Collapse pressure values of the order of 10^4 to 10^5 bar and collapse temperature values in the order of 5,000 K were observed. The collapse temperatures were seen to decrease on increasing driving frequencies.

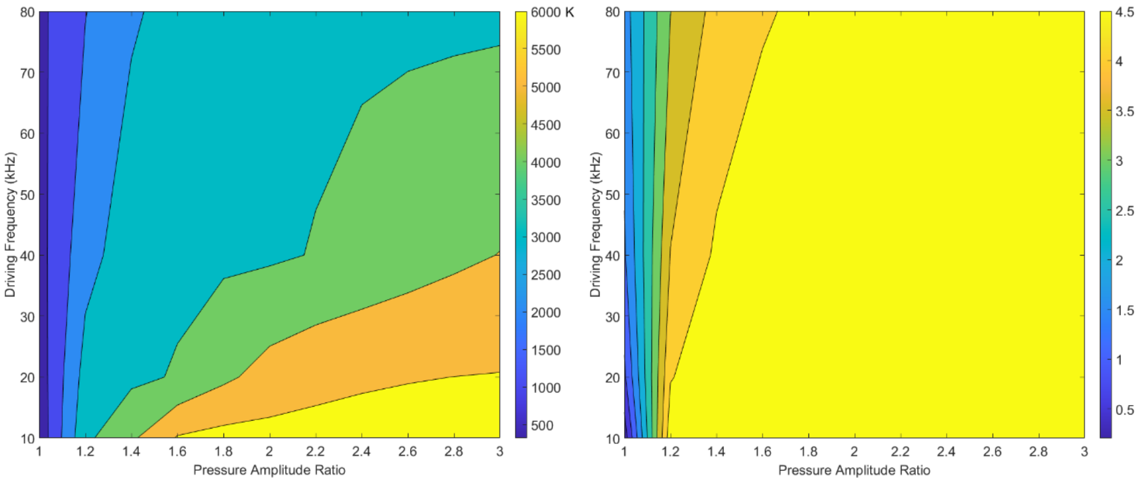

Figure A8.4: Comparison between the (a) collapse temperature (b) logarithm of collapse pressure versus P_A' profiles for varying values of driving frequency for acoustic cavitation

A8.5. Driving Frequency for Hydrodynamic Cavitation

The effects of the driving frequency on the peak pressures and temperatures for hydrodynamic cavitation are shown in Figure A8.5. Collapse pressure values of the order of 10^4 to 10^5 bar and collapse temperature values in the order of 5,000 K were observed. The collapse pressure and the collapse temperatures were seen to increase on decreasing driving frequencies. Notably, the collapse temperature for the case of 5kHz driving frequency was seen to be above (10,000 K) which was seen to have implications in terms of the OH radical formation discussed in Section 3.2.2.

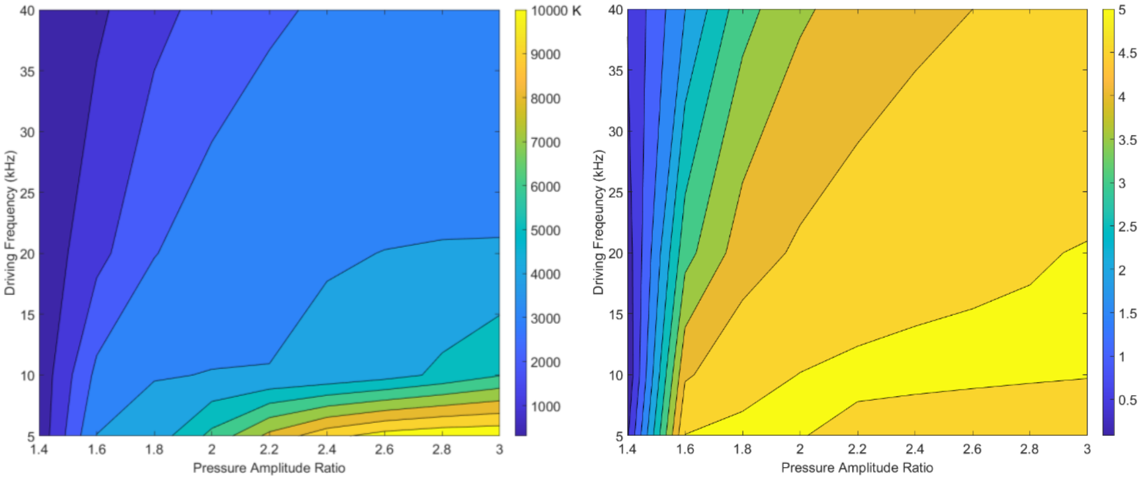

Figure A8.5: Comparison between the (a) collapse temperature (b) logarithm of collapse pressure versus P_A' profiles for varying values of driving frequency for hydrodynamic cavitation

A8.6. Initial Radius for Acoustic Cavitation

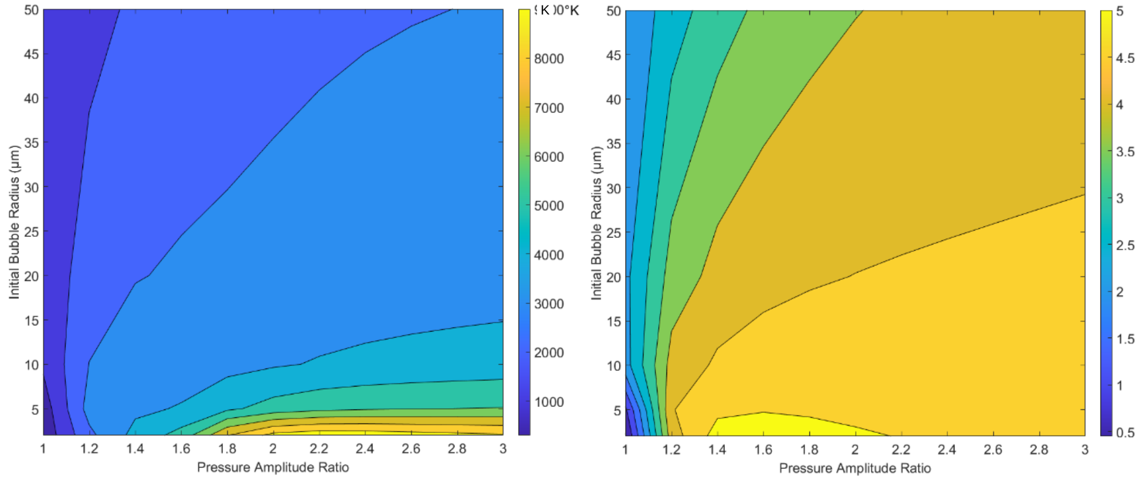

Figure A8.6: Comparison between the (a) collapse temperature (b) logarithm of collapse pressure versus P_A' profiles for varying values of initial bubble radius for acoustic cavitation

The effects of the initial radius on the peak pressures and temperatures for acoustic cavitation are shown in Figure A8.6. Collapse pressure values of the order of 10^4 to 10^5 bar and collapse temperature

values in the order of 5,000 K were observed. The collapse temperature was seen to decrease on increasing the initial bubble radius size.

A8.7. Initial Radius for Hydrodynamic Cavitation

The effects of the initial radius on the peak pressures and temperatures for hydrodynamic cavitation are shown in Figure A8.7. Collapse pressure values of the order of 10^4 to 10^5 bar and collapse temperature values in the order of 5,000 K were observed. Initially, if the bubble is very small, then the pressure oscillations due to a turbulent flow field may not be enough to induce cavitation and the bubbles would persist. These bubbles may coalesce to form larger bubbles which would then cavitate depending upon the threshold cavitation size for a given driving pressure amplitude. However, once the cavitation initiates, it has been reported that there is a strong attenuation of the pressure signal due to cavitation. Hence, the threshold size for cavitation would increase even further due to the attenuated pressure amplitude leading to increase in the cavity sizes and less intense cavitation events. Thus, the size of the cavities is a combined function of the threshold size required for initiating cavitation at a given pressure amplitude and the attenuated pressure amplitude due to the presence of the cavities. Simulations results suggest that the cavitation dynamics is more sensitive to the initial bubble radius for HC than for AC.

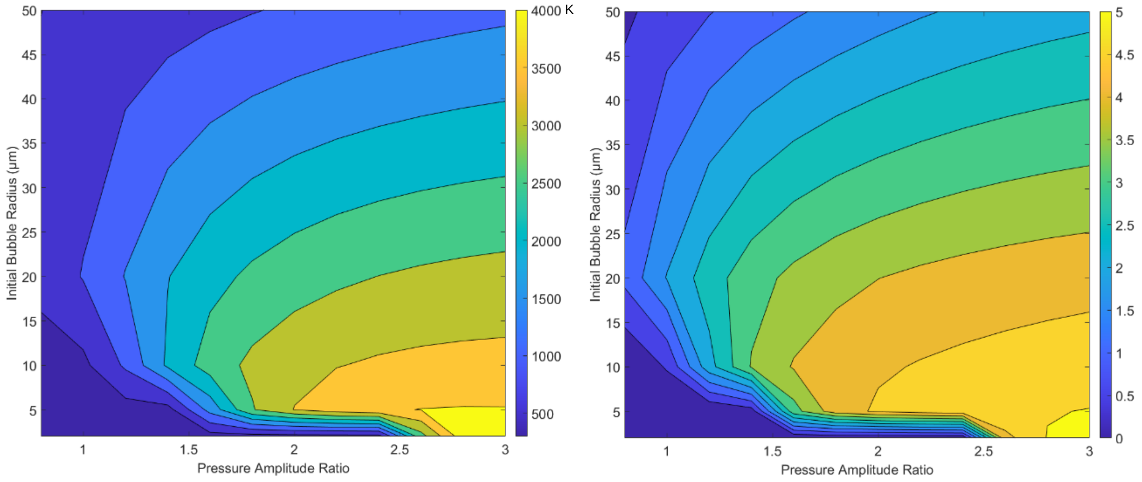

Figure A8.7: Comparison between the (a) collapse temperature (b) logarithm of collapse pressure versus P_A' profiles for varying values of initial bubble radius for hydrodynamic cavitation
